# From somatic variants towards precision oncology: Evidence-driven reporting of treatment options in molecular tumor boards

**DOI:** 10.1186/s13073-018-0529-2

**Published:** 2018-03-15

**Authors:** Júlia Perera-Bel, Barbara Hutter, Christoph Heining, Annalen Bleckmann, Martina Fröhlich, Stefan Fröhling, Hanno Glimm, Benedikt Brors, Tim Beißbarth

**Affiliations:** 1 Department of Medical Statistics, University Medical Center Göttingen, 37073 Göttingen, Germany; 2 Division Applied Bioinformatics, German Cancer Research Center (DKFZ) and National Center for Tumor Diseases (NCT), 69120 Heidelberg, Germany; 3 Division Translational Oncology, National Center for Tumor Diseases (NCT) and German Cancer Research Center (DKFZ), 69120 Heidelberg, Germany; 4 Department of Hematology and Medical Oncology, University Medical Center Göttingen, 37075 Göttingen, Germany; 5 German Cancer Consortium (DKTK), 69120 Heidelberg, Germany; 6 Department of Translational Medical Oncology, NCT-Dresden, University Hospital, Carl Gustav Carus, Technische Universität Dresden, 01307 Dresden and DKFZ, Heidelberg, 69120 Germany

**Keywords:** Genomic report, Personalized treatment, Cancer genomics, Targeted therapies, Predictive biomarkers, Molecular tumor board, Actionable variants

## Abstract

**Background:**

A comprehensive understanding of cancer has been furthered with technological improvements and decreasing costs of next-generation sequencing (NGS). However, the complexity of interpreting genomic data is hindering the implementation of high-throughput technologies in the clinical context: increasing evidence on gene–drug interactions complicates the task of assigning clinical significance to genomic variants.

**Methods:**

Here we present a method that automatically matches patient-specific genomic alterations to treatment options. The method relies entirely on public knowledge of somatic variants with predictive evidence on drug response. The output report is aimed at supporting clinicians in the task of finding the clinical meaning of genomic variants. We applied the method to 1) The Cancer Genome Atlas (TCGA) and Genomics Evidence Neoplasia Information Exchange (GENIE) cohorts and 2) 11 patients from the NCT MASTER trial whose treatment discussions included information on their genomic profiles.

**Results:**

Our reporting strategy showed a substantial number of patients with actionable variants in the analyses of TCGA and GENIE samples. Notably, it was able to reproduce experts’ treatment suggestions in a retrospective study of 11 patients from the NCT MASTER trial. Our results establish a proof of concept for comprehensive, evidence-based reports as a supporting tool for discussing treatment options in tumor boards.

**Conclusions:**

We believe that a standardized method to report actionable somatic variants will smooth the incorporation of NGS in the clinical context. We anticipate that tools like the one we present here will become essential in summarizing for clinicians the growing evidence in the field of precision medicine. The R code of the presented method is provided in Additional file 6 and available at https://github.com/jperera-bel/MTB-Report.

**Electronic supplementary material:**

The online version of this article (10.1186/s13073-018-0529-2) contains supplementary material, which is available to authorized users.

## Background

Precision medicine (PM) aims at understanding, and thus managing, a patient’s disease based on its molecular profile. In oncology, PM is being implemented through tumor boards (TB), multidisciplinary meetings where patients are discussed from all possible perspectives (diagnostic radiology, surgery, pathology, genetics, oncology, radiotherapy, etc.). Currently, TB include routine testing of several predictive biomarkers which indicate the likelihood to respond to a specific treatment. However, these are restricted to few cancer entities and just a handful of examples exist, for instance EGFR inhibitors in non-small cell lung cancer tumors with *EGFR* mutations in exons 19/21 (*afatinib*, *erlotinib*, *gefitinib*) or resistance mutation T790 M (*osimertinib*), HER2 inhibitors (*trastuzumab*) in breast tumors with *HER2* amplification/overexpression, and BCR-ABL inhibitors (*bosutinib*, *desatinib*, *imatinib*, *nilotinib*, *ponatinib*) in Philadelphia chromosome-positive hematologic malignancies [1]. Predictive biomarkers are recognized by reference approval and guideline organizations (e.g., The Food and Drug Administration (FDA), The European Medicines Agency, and the National Comprehensive Cancer Network (NCCN)) and are specific for a cancer type. When a tumor progresses after the regulated, evidence-based therapies have been applied, testing for genomic alterations of targets of drugs approved for other cancer entities, or even drugs under clinical trials, becomes a common strategy for rational prescription of drugs. However, even if a drug target harbors a mutation, it does not mean that the variant predicts drug response. Inferring causality between a gene variant and a drug response in an *off-label* scenario (e.g., another cancer type, unknown mutation in the target gene, and/or mutation in a gene upstream of the target gene) is very challenging [2]. For this reason, the scientific community has already stated the need for a comprehensive knowledge database of variants and affected genes with respect to drug response (i.e., actionable variants) [3]. In the same direction, the clinical community needs decision support platforms for interpreting somatic alterations in regard to clinical action [4–7]. Last, but not least, there is a gap between legislation and health insurance policies with regard to who should pay for these expensive treatments in *off-label* scenarios. Typically, health insurance companies will only pay for these treatments if the clinician is able to prove causality. Otherwise, the only opportunity to receive an *off-label* treatment will be under the umbrella of a clinical trial. In turn, clinical trial enrollment can be complex due to strict inclusion criteria (e.g., restricted to some cancer entities, molecular markers, and previous therapies) and location of the clinical facilities.

In addition, the latest advances in next generation sequencing (NGS) will, in the foreseeable future, allow us to sequence the whole genome of patients in a reasonable time. Few worldwide clinical institutions have taken the first steps in this direction by implementing molecular tumor boards (MTBs) to study the feasibility of performing comprehensive genomic profiling (e.g., NGS) in the clinical context [8–16]. However, NGS generates huge volumes of data, ranging between 5 and 200 Gb per patient, depending on coverage, sequencing technique (whole exome, whole genome), etc. Hence, interpreting the clinical implications of single nucleotide variants (SNVs), copy number variations (CNVs), and gene fusions of a tumor biopsy (i.e., somatic variants) becomes increasingly complex. One main difficulty lies in distinguishing the relevant alterations with either diagnostic, prognostic, or predictive meaning from all the others [3, 17]. Compiling a comprehensive list of somatic variants for one patient, scanning through all the variants, and summarizing the clinical implications and evidence based on current literature knowledge is already a huge task. For that, clinicians integrate information from web-based tools such as *My Cancer Genome* (mycancergenome.org), *OncoKB* [18], or literature searches (*PubMed*, *Google Scholar*). Nevertheless, this strategy is far from being optimal: it requires time, is subject to omission of information, and depends on the clinician’s ability to interpret the clinical implications from genomic research articles. Recent studies have predicted that the use of NGS profiling and drug *repurposing* have the potential to identify actionable genomic alterations in more than 70% of cancer patients [19, 20]; however, the clinical reality is very far from this.

In this work, we aim to generate an automated tool that produces patient-specific reports comprising a filtered list of patient’s actionable variants with respect to potential treatment options. Although treatment decisions will always require expert knowledge, we hypothesize that such a tool will substantially facilitate the use of NGS in clinical practice by simplifying the task of finding the clinical implications of genomic variants. To this end, we present here a framework that pre-filters, classifies, and reports actionable variants, defined as somatic genomic alterations, which have some evidence of drug response or resistance. We then show the results of applying the tool to different patient cohorts (The Cancer Genome Atlas (TCGA), Genomics Evidence Neoplasia Information Exchange (GENIE), and NCT MASTER). With it, we hope to provide a proof-of-concept of a tool that can filter a list of variants and link them to available evidence for genomic-based treatment decisions (e.g., information from literature, clinical trials, and databases).

## Methods

In this article we present a framework to match tumor genomes to targeted therapies. For that, we use public databases to filter potentially actionable variants from a tumor sample and then classify the results using an evidence-based system. In this section we first detail the databases used. Then, we present the patients’ datasets used for evaluating the feasibility and provide a proof-of-concept of our approach. Finally, we describe all statistical analyses.

### Databases of actionable variants

Among the main criteria of database selection, we focused on those databases that compile information not only on the drug and actionable gene, but also the actionable variant (SNV, CNV, rearrangement), the type of association (response, resistance), the strength of the evidence (approved, clinical trials, preclinical) and the cancer type. As a result, we have selected the following databases: (1) *Gene Drug Knowledge database* (GDKD) [20] (version 19, downloaded from Synapse syn2370773); (2) *Clinical Interpretation of Variants in Cancer* database (CIViC) [21] (version 01_June_2017); and (3) *Tumor Alterations Relevant for Genomics-driven Therapy* database (TARGET) [12] (version 3). GDKD is a manually curated database of predictive biomarkers updated monthly. It integrates several layers of annotations, comprising cancer type, gene, variant, response/resistance, consensus/emerging, and the corresponding references. CIViC uses very similar layers of annotation, but is a community-driven web resource. For this work, both GDKD and CIViC were modified in such a way that variants in the same gene sharing annotations of disease, drug, evidence, and association levels were aggregated into one single entry. Finally, TARGET was published in 2014 and has not been updated since. Taken together, the three databases compile a comprehensive list of variants comprising a total of 289 actionable genes (conferring either resistance or response to anticancer drugs). The main characteristics of the three databases can be found in Table 1. We also consulted other sources such as NCCN guidelines, mycancergenome.org, and Meric-Bernstam et al. [22] and manually added some expert rules. The complete list of 312 actionable genes can be found in Additional file 1.

**Table 1:** Main characteristics of the public databases of predictive biomarkers

		GDKD	CIViC	TARGET
Number of genes		170	290	135
Number of predictive genes	Total	170	213	111
	Exclusive	46	105	10
	Common	79		
Number of variant–drug associations		618	1931	111
Number of cancer types		65	177	Not specified
Biomarker types		Predictive	Predictive	Predictive
			Prognostic	Prognostic
			Diagnostic	Diagnostic
Clinical significance levels		Response	Sensitivity	Free text
		Sensitivity	Resistance or non-response	
		Increased benefit		
		No response		
		No sensitivity		
		Reduced/decreased sensitivity		
		Resistance		
Evidence levels		NCCN/FDA	A: clinical routine	None
		Late trials	B: clinical trials	
		Early trials	C: case reports	
		Case report	D: preclinical	
		Preclinical	E: inferential	
Variant specific		Yes	Yes	No
References provided		Yes	Yes	No
Version		v19	1 June 2017	v3
Source		[20]	[21]	[12]

Each column summarizes the specificities of each database: *GDKD* Gene Drug Knowledge Database, *CIViC* Clinical Interpretation of Variants in Cancer, *TARGET* Tumor Alterations Relevant for Genomics-driven Therapy

### Datasets

#### TCGA and GENIE datasets

The *Pan Cancer 12* data freeze of TCGA was used as a patient cohort for testing our reporting method. Three data types were downloaded from the Synapse repository: clinical data (syn2325436), somatic SNVs (syn1729383), and somatic CNVs (syn1711454). Data on a total of 5277 samples from 12 different cancer types were collected. Only samples with both mutation and copy number data (3184 samples) were considered for the analyses in this paper. Regarding mutation data, “silent” variants were not studied. For CNVs, the output from GISTIC 2.0 *all_thresholded.by_genes* was used. This file contains the copy number data of genes discretized into values of the set {− 2, − 1, 0, 1, 2}, where 0 means no deletion or amplification, +/− 1 means amplification or deletion above the low noise threshold and +/− 2 are amplifications and deletions above the high level threshold. High level thresholds are calculated on a sample basis and are an approximation to homozygous events. Only high level amplifications and deep losses (+/− 2) were considered for the analysis, as previously done [23]. The cancer type abbreviations used throughout the article and number of samples are: BRCA (breast cancer, 756), BLCA (bladder cancer, 97), UCEC (uterine cancer, 244), READ (rectal cancer, 69), COAD (colon cancer, 155), OV (ovarian cancer, 313), LUSC (lung squamous carcinoma, 178), LUAD (lung adenocarcinoma, 172), LAML (acute myeloid leukaemia, 190), KIRC (kidney cancer, 417), HNSC (head and neck cancer, 306), and GBM (glioblastoma multiforme, 287).

The GENIE dataset was downloaded from the Synapse repository (SNVs, syn7851250; CNVs, syn7851245; fusions, syn7851249; clinical data, syn7851246). This dataset comprises data of 18,804 advanced cancer patients with more than 50 different cancer entities [24]. Mutation and copy number data were analyzed in the same way as described for TCGA dataset.

### NCT MASTER dataset

#### Patients

A proof-of-concept of the method in a clinical context was investigated within a retrospective study. We used the data of 11 patients with advanced tumor diseases who had undergone whole exome and transcriptome sequencing within the so-called NCT MASTER trial, an institutional review board-approved clinical sequencing program for young adults with advanced-stage hematological and oncological diseases across all malignancies. A tumor tissue and a matched normal blood sample for whole-exome sequencing were obtained following written informed consent under an institutional review board-approved protocol.

#### Whole-exome sequencing data

Tissue samples were provided by the NCT Heidelberg Tissue Bank. Whole-exome sequencing of normal and tumor tissue samples was followed by a bioinformatic analysis for detecting SNVs, small insertions and deletions (indels), CNVs, and structural variations that might lead to gene fusions. On average, coverage was 133× and 126× for tumor and normal samples, respectively (sample-wise data can be found in Additional file 2). Reads were mapped to the 1000 Genomes phase 2 assembly of the human reference genome (NCBI build 37.1) using BWA (version 0.6.2) with default parameters and maximum insert size set to 1000 bp [25]. BAM files were sorted with SAMtools (version 0.1.19) [26] and duplicates were marked with Picard tools (version 1.90). For the detection of SNVs, we applied our in-house analysis pipeline based on SAMtools mpileup and bcftools with parameter adjustments to allow the calling of somatic variants with heuristic filtering as previously described [27–29]. We used Platypus [30] version 0.5.2 to identify indels with a similar reliability scoring as for SNVs. All mutations were annotated with ANNOVAR [31] version September 2013 using the RefSeq gene model. From the set of somatic high confidence mutations, we extracted nonsynonymous, stopgain, and stoploss SNVs as well as SNVs at splice sites, and indels that are located in a coding sequence or splice site. CNVs were analyzed by read depth plots and an in-house pipeline using the VarScan2 copynumber and copyCaller modules [32]. Regions were filtered for unmappable genomic stretches and merged by requiring at least 70 markers per called copy number event. We selected regions with a log ratio of tumor coverage over control coverage higher than 0.55 or lower than − 0.55 as copy number gains and losses, respectively, and annotated them with RefSeq genes using BEDTools [33]. We searched for structural variants such as translocations that might lead to gene fusions with CREST [34] on the DNA level.

The variant calls were delivered in excel files, and for each patient, a group of expert bioinformaticians and oncologists manually revised the list of somatic alterations looking for actionable alterations that could guide the treatment decision. The genomic somatic calls of the patients, together with the experts’ interpretations, are summarized in Additional file 2.

### Statistical analyses

We performed an unsupervised clustering using the molecular status of the 312 actionable genes in the 3184 TCGA tumor samples. Four molecular categories were used: wild type, mutated, high-level amplification, and deep loss. Genes with no mutations or with “silent” variants were considered as wild type. Genes with any other type of mutation were considered as mutated. With respect to CNVs, GISTIC output *all_thresholded.by_genes* was used*;* genes with a value of − 2 were classified as deep losses and + 2 as high-level amplifications. Next, our algorithm to filter variants was applied, and a molecular status matrix (sample x gene) was constructed. Genes without any alteration in any sample were removed. Complete-linkage hierarchical clustering was performed on rows and columns based on the Gower distance metric for nominal data (*daisy* function from R package *cluster*). The respective heatmap of all samples and the top 50 genes was plotted with dendrogram on the columns (*heatmap.2* from *gplots* R package).

## Results

In this section, we first present the framework to filter, classify, and report actionable genomic alterations to support treatment decisions. We then present the results of applying the tool to three patient cohorts with the aim to: 1) evaluate its feasibility with the TCGA and GENIE cohorts and 2) provide a proof-of-concept of the reports with the NCT MASTER cohort.

### Reporting framework

**Fig. 1:**
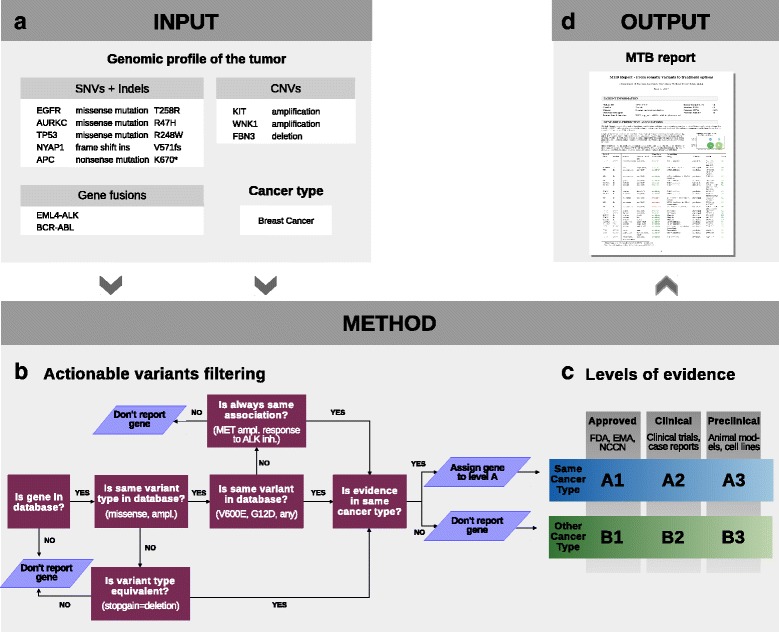
Overview of the pipeline to report actionable variants from tumor genomic profiles. **a** The algorithm uses two types of input: type of tumor (e.g., breast cancer) and its genomic profile (i.e., somatic variants). **b** First, the genomic profile is used to identify the actionable variants as depicted in the flowchart. A variant with an established significance will follow the central path of the flowchart (e.g., *BRAF* V600E). The side arms are designed to *repurpose* variants of unknown significance. **c** Then, the actionable variants are classified into clinically relevant categories using a system of six levels of evidence. **d** Finally, the output is in form of hand-in reports

**Fig. 2:**
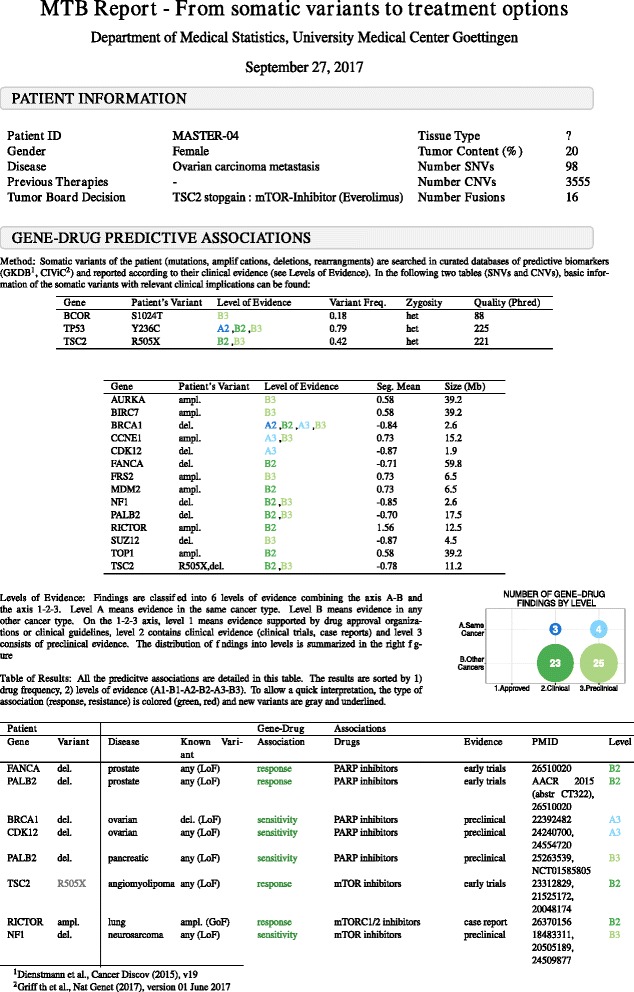
The molecular tumor board (*MTB*) report. First page of the report of patient MASTER-04 from the NCT MASTER dataset. General information of the patient, clinical history, and genomic data are summarized under a first header entitled “Patient information”. Under a second block called “Gene-drug predictive associations”, the user can find all the details regarding the actionable variants identified. The method is briefly described at the beginning. The number of gene–drug predictive associations found at each level are summarized in a figure and then detailed in a table. In the table, the patient’s variants are located in the left part, and the public knowledge on those variants is located in the right part. Each row details a specific association between a gene variant and a drug response in a specific cancer type

In order to exemplify the steps of the framework (Fig. 1), we will use patient MASTER-04 from the NCT MASTER dataset. This patient presented with metastatic ovarian carcinoma, and, after whole-exome sequencing, the panel of experts of the NCT MASTER suggested *everolimus*, an mTOR inhibitor, as the best treatment option based on a stopgain mutation in the gene *TSC2* (Fig. 2).

#### Filtering of actionable somatic variants

Two types of information are required as input (Fig. 1a): cancer type (e.g., breast cancer) and the somatic variants. Three kinds of somatic variants can be used as input: SNVs and indels, CNVs, and fusion genes. In patient MASTER-04, 98 missense SNVs, 16 fusion genes, and CNV regions (focal and broad) comprising 3555 genes were identified by the bioinformatic pipeline and used as input for the tool. The method assumes that any quality thresholds are previously applied. Therefore, it assumes that only high quality variants are used as input.

In general, variants are annotated in the databases in two different ways: 1) as hotspot mutations/CNVs/fusions with very well-known therapeutic implications (e.g., T790 M or exon 19 mutations in *EGFR*, *HER2* amplification in breast cancer); or 2) as “any missense mutation” or just “any loss/gain of function” variant in those cases where there is no hotspot mutation known or all variants within a gene are studied (very common in clinical trials and preclinical studies).

To identify actionable variants, the algorithm follows a narrowing down procedure. First, patient variants are queried at gene level. Then, for each altered gene, the patient variant type is matched to database entries (e.g., missense mutations, copy number loss, copy number gain). In case of SNVs, the protein change is further checked. If the patient protein change is present in the database, or if the gene annotation is “any missense mutation”, the variant is retained for further steps. If not, the variant is considered of unknown significance and *repurposing* rules are applied; a variant of unknown significance will be matched to hotspot mutations on the same gene if all the associations between that gene and the drug on the database are in the same direction (i.e., always in favor of response to a drug, or always indicating resistance). The algorithm will also match a stopgain mutation in a gene “with copy number loss/loss-of-function” entries in the database (Fig. 1b). In patient MASTER-04 we can see an example of such a *repurposing* rule: *TSC2* stopgain mutation (R505X) is identified as a loss-of-function mutation (Fig. 2).

#### Classification and reporting: evidence-based MTB report

After filtering, the algorithm classifies each variant–drug association according to their evidence. The goal is to include additional options for *off-label* use that may be applicable for advanced-stage patients who failed all conventional standard therapy. To this end, we have created a six-level system to rank the associations according to their evidence. This system allows a stratification of possible treatments into two axes: strength of clinical evidence (axis 1, 2, 3) and cancer type (axis A, B) (Fig. 1c). Level A means evidence in the same cancer type. Level B means evidence in any other cancer type. On the 1-2-3 axis, level 1 means evidence supported by drug approval organizations or clinical guidelines. Level 2 contains clinical evidence, in which late clinical trials are ranked higher followed by early clinical trials and case reports. Finally, level 3 consists of preclinical evidence.

The output of this algorithm is a report designed as a tool to facilitate genomic-based treatment decisions in MTB (Figs. 1d and 2) by compiling all actionable variants of a patient. The quality of the variants is summarized in two tables (SNVs and CNVs separately). The number of variant–drug associations at each level is depicted in a figure. The results are detailed in a table ranked by 1) drug frequency (number of times the drug appears associated with response), 2) by levels of evidence (A1 > B1 > A2 > B2 > A3 > B3) and 3) by gene. This way of sorting aims at ranking first the therapeutic options with more support, but at the same time with high evidence (in patient MASTER-04, we see that mTOR inhibitor is the treatment with the highest number of results and, hence, it is ranked as the first option). Also, by grouping the drugs we allow a better visualization of 1) contradicting evidence (resistance and response to the same drug) and 2) different variants supporting the same treatment. To allow a quick interpretation, the type of association (response, resistance) is colored (green, red). The variants of unknown significance are gray.

### Scope of the MTB report

To obtain cohort-level information on automatically generated recommendations, we applied our method to 3184 samples of the *Pan Cancer 12* dataset from TCGA and to 18,804 samples of GENIE.

#### Distribution of actionable variants in the TCGA cohort

**Fig. 3:**
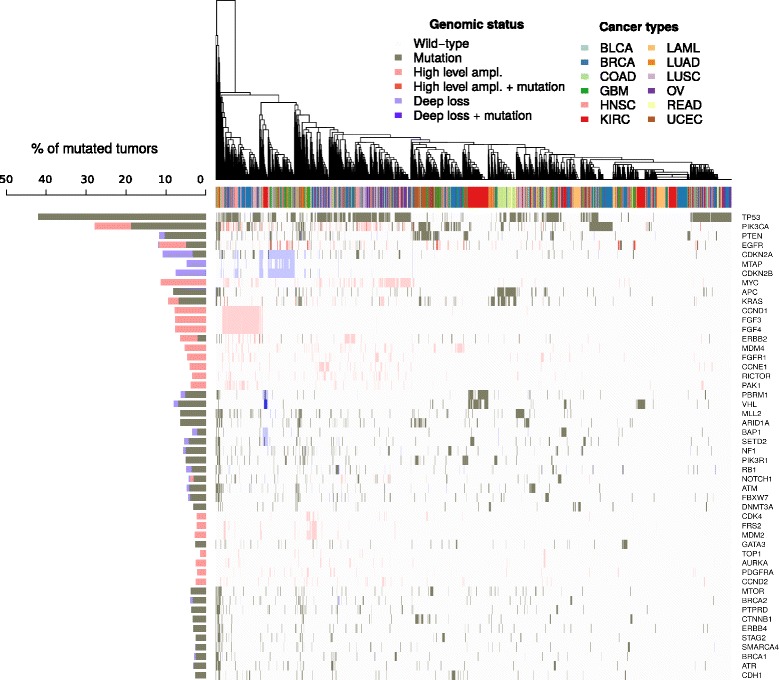
Unsupervised clustering of 3184 TCGA samples based on genomic status of 312 genes. The figure displays a heatmap of the genomic status of the top 50 most altered genes (*rows*) on 3184 tumor samples (*columns*) with dendrogram from hierarchical clustering of the samples. The percentage of mutated samples of every gene is vertically displayed at the *left* of the heatmap (histogram). The legend *Cancer types* refers to the annotation of the tumor samples in the columns, the legend *Genomic status* describes the colors used in the heatmap and the histogram

In order to determine the feasibility and extent of incorporating genomic data into clinical decisions, we first identified the actionable variants of every sample. We performed an unsupervised clustering of the cohort using the molecular status (i.e., wild type, mutated, amplified, deleted) of the variants found by the algorithm (Fig. 3). The clustering did not reveal any histology-specific molecular signature. In other words, we did not observe specific sets of known biomarkers defining cancer types.

Overall, samples with none or few genomic events clustered at the right side of the dendrogram, comprising a subset of acute myeloid leukemia, kidney, and breast cancer samples. On the other extreme of the clustering we found a subset of hypermutated samples of colorectal and endometrial cancers. In between, biomarkers were altered across many histologies in low frequencies rather than being histology-specific. However, some exceptions could be observed: *TP53* and *PIK3CA* were mutated in ~ 30–40% of the samples, *FGFR3/4* were almost exclusively amplified in a subgroup of breast and squamous malignancies (head and neck and lung squamous cancers), and *CDKN2A/B* deletions clearly defined a subgroup of glioblastoma samples mixed with lung and head and neck samples. The two latter examples are interesting since clinical trials for these biomarkers exist for breast and glioblastoma tumors, respectively. However, according to these results, *FGFR3/4* and *CDKN2A/B* could be biomarker candidates to be studied on lung and head and neck cancers.

Finally, for this cluster analysis 156 genes (out of 312) were removed due to the lack of alterations in any samples. The fact that half of the biomarkers are not present in a cohort of 3184 samples highlights the low frequency of some genomic events and the problem that this poses for biomarker validation. Of course, the tissue representation of this cohort has also an important impact on the alterations found. All in all, the results support the principle of performing comprehensive molecular characterization in clinical practice instead of only testing a histology-specific panel of biomarkers.

#### Impact of genomic-guided treatment options on TCGA cohort

**Fig. 4:**
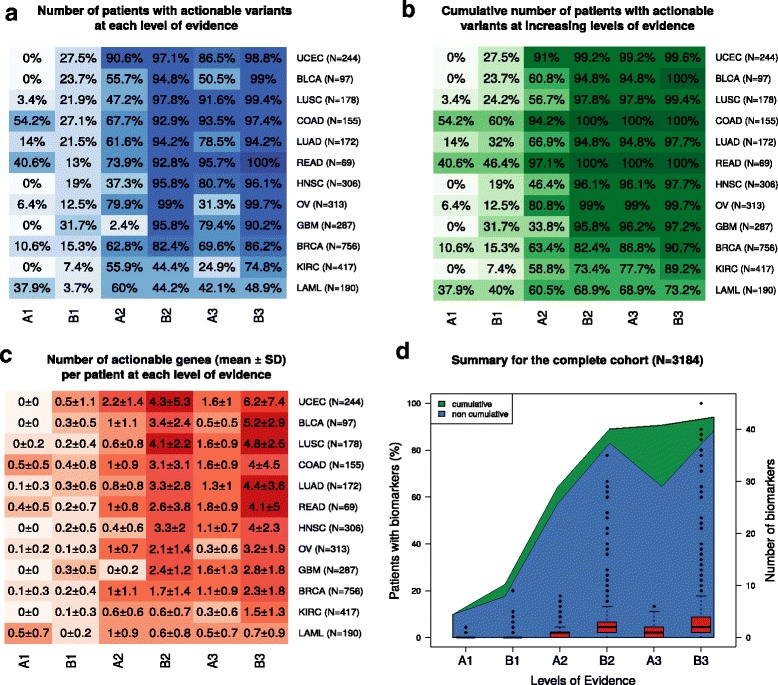
Heatmap representation of the distribution of identified actionable variants in TCGA cohort. Somatic alterations of each sample (3184 samples) were analyzed as depicted in Fig. 1 and the resulting biomarker–drug associations were assigned to one of the six levels of evidence. Evidence in wild-type variants and resistances are not included in this representation (unless if the evidence is in level A1, e.g., *NRAS*, *KRAS* wild type in colorectal cancer). **a** The percentage of patients with at least one actionable variant at each level of evidence. **b** The cumulative percentage of patients with at least one actionable variant at increasing levels of evidence (from A1 to B3, *x-axis*). **c** Average (± standard deviation (*SD*)) number of actionable genes per patient at each level of evidence. **d** Combination of the data shown in **a**–**c** panels depicted for the whole cohort, no distinction among cancer types

Following the identification of actionable variants, the variant–drug pairs of each patient were classified into one of the six levels of evidence, as described in the “Reporting framework” section and Fig. 1. The results of this analysis allowed us to evaluate the performance of our method at suggesting treatment options. We identified the fraction of patients that received treatment suggestions at each level of evidence independently (Fig. 4a), and cumulatively from the highest evidence to the lowest (A1 > B1 > A2 > B2 > A3 > B3) (Fig. 4b). We could also determine the number of genes with actionable variants identified at each level (Fig. 4c). Finally, these three measurements are depicted in Fig. 4d for the entire cohort.

The algorithm found actionable variants with supporting evidence for drugs approved for the underlying entity (level A1) in 9.9% of the investigated cancer patients. A1 actionable variants were found in only half of the cancer types, and, among these, two main groups could be differentiated: 1) colon, rectal, and leukemia (> 30% of patients) and 2) lung squamous, lung adenocarcinoma, breast and ovarian cancer (< 15% of patients) (Fig. 4a, b). Of course, there may be further A1 actionable variants in the cohort that we cannot identify at the DNA level (e.g., Her2neu or CD20 protein expression) and because we did not include fusion data in this analysis (e.g., *ALK* fusion in lung adenocarcinoma).

Nevertheless, the percentage of patients with actionable variants doubled (22.7%) when we considered also level B1—drugs approved for the treatment of other cancer types (Fig. 4d). This rise was due to alterations at a low percentage in genes with proven predictive value in the context of approved anti-cancer drugs (e.g., *EGFR*, *BRAF*, *ERBB2*, *BRCA1*, *BRCA2*, *RET*, *ALK*) in all cancer types (Additional file 3). Kidney cancer as well as acute myeloid leukemia overall tended to harbor fewer actionable mutations (Fig. 4c), in which the incidence of B1 actionable variants reached less than 10% of patients (Fig. 4a), pointing towards different molecular mechanisms driving these two cancer types. As for breast cancer, there was almost 80% overlap of patients between A1 and B2 levels (Fig. 2b). The reason for it is that they are based on the same alteration: *HER2* amplification is an approved biomarker for *trastuzumab*, *lapatinib*, *pertuzumab* in breast cancer (A1) as well as for *trastuzumab* in gastric cancer (B2).

The most significant increase in the ratio of patients with actionable alterations happened when considering drugs in clinical trials (A2), which reached 64.1% of patients (Fig. 4d). In this context, the number of patients with variants studied in clinical trials was significantly lower in glioblastoma and kidney cancer when compared to other entities (Fig. 4a, A2 level). Clinical trials on other cancer entities (B2) further increased the coverage to 89% of patients. Whereas at the B2 level the increase seems to be correlated to a higher number of different biomarkers included in clinical trials, this is not the case for the A2 level (Fig. 4c, d). Additionally, kidney cancer and acute myeloid leukemia still remained different at the B2 level (43.6 and 38.4%, respectively, vs > 80%; Fig. 4a). Finally, including preclinical evidence (A3, B3) did not have a further impact on the range of patients with tumors harboring actionable alterations (Fig. 4b, d).

We compared our results with previous *in silico* efforts to characterize the actionable landscape of TCGA cohort (Table 2). These studies used different subsets of TCGA cohort and also different databases to identify actionable variants. For these reasons, the results are not directly comparable. However, reporting lower-evidence biomarkers (i.e., *off-label* scenarios and on substances in clinical trials) undeniably increased treatment recommendations for cancer patients in all studies. Also, there were clear similarities in the different classification systems used. The study from Dienstmann and colleagues [20] presented the most similar results to ours, most likely because our method uses, among others, their database.

**Table 2:** TCGA actionable landscape in different publications

	Standard therapy		Clinical trials		Preclinical		Total	Number of TCGA samples	Databases used by the study
	Label	Off-label	Label	Off-label	Label	Off-label			
MTB	9.9 (A1)	22.7 (B1)	64.1 (A2)	89 (B2)	90.6 (A3)	94.1 (B3)	94	3184	GDKD, CIViC, TARGET
Dienstmann et al. 2015 [20]	11 (5)	–	39 (4)	75 (3)	93 (1-2)		93	4392	GDKD
Rubio-Perez et al. 2015 [19]	5.9	40.2	73.3		–	–	73.3	4068	Rubio-Perez et al. 2015
Chakravarty et al. 2017 [18]	7.5 (1–2A)	16 (2B)	26 (3A)	41 (3B)	–	–	41	5983	OncoKB

The table shows the cumulative percentages of patients with actionable variants identified at different levels. The name of the levels used in each publication are specified in parentheses. The studies being compared are: MTB (this publication), Chakravarty et al. 2017 [18], Dienstmann et al. 2015 [20], and Rubio-Perez et al. 2015 [19]

#### Impact of genomic-guided treatment options on the GENIE cohort

The same analysis was performed on 18,804 samples of the GENIE cohort (Additional file 4). This cohort is especially interesting because of a higher proportion of advanced tumors—compared to TCGA—and because of the targeted screening for cancer-relevant genes—panels covering from ~ 50 up to ~ 450 genes. Our method identified A1 actionable variants in 15.3% of the samples. Compared to TCGA cohort, main differences could be observed in non-small cell lung cancer and leukemia in the A1 level. In B1, GENIE cohort showed a clearly higher percentage of patients with actionable alterations (38.4%). This is not surprising since these patients have acquired mutations during the course of the disease (*EGFR* T790 M) and the fact that this dataset includes fusions (*ROS1*, *ALK* in lung cancer, *ABL1* in leukemia). However, the remaining levels of evidence shared similar ranges in both datasets, being even a bit superior in TCGA cohort. This is most likely due to the fact that TCGA covered exome-wide analysis whereas GENIE is restricted to a panel of genes.

### Retrospective evaluation of the MTB report on 11 advanced cancer patients

As a proof-of concept of our reporting method (MTB report), we performed a retrospective study on 11 patients with advanced malignancies. Whole exome sequencing of normal and tumor tissue samples was performed within the NCT MASTER program, a clinical sequencing program including young adult cancer patients, followed by bioinformatic analysis for detecting SNVs, indels, CNVs, and fusions (see “Methods”). For each patient, a group of experts (bioinformaticians and oncologists) manually revised the list of somatic alterations looking for variants that could guide the treatment decision. The selected actionable alterations were discussed in the NCT MASTER program molecular tumor board. In case of many variants in the same tumor, only the one to three most relevant findings were discussed. We have compared the alterations discussed by the experts in the NCT MASTER program (MASTER report) with the results reported by our method (MTB report). The 11 MTB reports generated with our tool can be found in Additional file 5.

The MTB reports showed a very good performance in terms of sensitivity in the detection of actionable variants: in 10 out of 11 patients, mostly all variant–drug associations in the MASTER report matched our MTB report (Table 3). Overall, only four genes suggested in the MASTER reports were not detected by our tool: *PTPRJ*, *PTPN12*, *LCK*, and *NTRK3*. The first three are not present in any of the databases used by our method. As for *NTRK3*, it is present in GDKD as a gene fusion biomarker. However, the patient presented a missense mutation in this gene, and our tool does not allow *repurposing* of fusions into missense mutations to avoid too many false positive results.

**Table 3:** Retrospective comparison in 11 patients

New ID	Cancer	Number of SNVs	Number of CNVs	MASTER report		MTB report			
				Gene	Drug	Number of results	Match	Match level	Drug support
MASTER-01	Breast cancer metastasis	104	3410	*BRCA1/2* deletions	PARP inhibitors	43	Yes	B2	11.6%
				*RAF1*, *PDGFRA* amplifications	Sorafenib		Yes	B3	4.6%
				*FGF1* (T8N)	FGFR inhibitor		Yes	–	2.3%
MASTER-02	Pancreatic adenocarcinoma	49	1528	*KRAS* (G12D)	–	92	–	–	–
MASTER-03	Leiomyosarcoma of the retroperitoneum	31	3181	*PTPRJ* deletion	Pazopanib	12	No	–	–
				*CDK12* + *BRCA2* deletions	Cisplatin		Yes	B2	50%
MASTER-04	Ovarian carcinoma	98	3385	*TSC2* (R505X)	mTOR inhibitor	56	Yes	B2	12.5%
MASTER-05	Myxoid liposarcoma	11	72	*PIK3CA* (C420R) + *PTEN* (R130G)	mTOR inhibitor	75	Yes	B2	9.3%
					AKT inhibitor		Yes	B2	2.4%
					PI3K inhibitor		Yes	B2	32%
MASTER-06	Neuroendocrine tumor	2703	2060	*MTOR* (P2490L, G332R)	mTOR inhibitor	89	Yes	B2	16.8%
				*PTPN12* (S509 N, G523S)	Lapatinib, erlotinib, imatinib, desatinib		No	–	–
				*KIT* (A837T)	Imatinib, desatinib		Yes	B3	1.1%
				*LCK* (P74L)	Desatinib		No	–	–
MASTER-07	Neuroendocrine tumor	645	941	*ERBB3* (V104 M), *RAF1* (S259P), *MTOR* (E1485G)	mTOR inhibitor	40	Yes	A2	12.5%
MASTER-08	Cholangiocarcinoma	28	1001	*ERRFI1* (R199X)	Erlotinib	17	Yes	–	0.58%
MASTER-09	Clear cell sarcoma	11	1	*NTRK3* (R116W)	Lestaurtinib, midostaurin	3	No	–	–
MASTER-10	Histiocytic sarcoma	7	5	*BRAF* (F595 L) + *HRAS* (Q61R)	MEK inhibitor	11	Yes	B2	33.3%
					sorafenib (multi TKi)		Yes	B3	5.5%
MASTER-11	Pulmonary adenocarcinoma	70	146	*EGFR* (p.745-750del)	Erlotinib	54	Yes	B2	22.2%

Actionable variants discussed by experts as part of the NCT MASTER trial (MASTER report) were compared to actionable variants reported by our method (MTB report)

The majority of actionable variants in the MASTER reports were found in low evidence levels of the MTB report, mostly B2 and B3 levels. This agrees with the distribution of actionable events in TCGA cohort. Furthermore, the patients enrolled in this program are advanced cancer patients, meaning that they were heavily pretreated and did not have standard therapies left; hence, we would not expect A1 findings.

The actionable variants reported in the MTB report were redundant in terms of gene–drug associations; yet, they provided unique information regarding cancer type or clinical evidence (A, B, C). Indeed, the more times a drug was reported, the more evidence supported the eligibility of that drug (see “Drug support” percentages in Table 3). However, mutations in controversial genes and pathways may also generate conflicting results (e.g., *KRAS* mutation, see report MASTER-02 in Additional file 5), demonstrating the need for a multidisciplinary discussion of NGS data in the clinical context within MTBs.

Alterations of DNA repair pathway genes (*BRCA1*, *BRCA2*, *RAD51*, *PALB2*, *CDK12*) predicted response to PARP inhibitors in several MTB reports. Based on the concept of synthetic lethality upon an initial disruption on DNA repair, MASTER reports included platin-based chemotherapies whenever this pathway was mutated. Therefore, these two therapies were considered equivalents and assessed as a “match” in Table 3.

## Discussion

Despite rapid advances in high-throughput screening technologies, only a handful of predictive biomarkers are routinely tested in TB to guide treatment decisions (e.g., *EGFR* in non-small cell lung cancer or *KRAS* in colorectal cancer). Some clinical institutions are now starting to implement panel sequencing for some tumor entities (e.g., lung cancer and myelodysplastic diseases). Moreover, several clinical studies have anticipated the feasibility of using NGS for treatment decisions, although its impact on patient outcome is still under discussion [8–11, 13, 16]. However, the high dimensionality of NGS data together with the growing evidence of biomarkers for targeted therapies makes the task of identifying actionable somatic variants time-consuming. In this work, we present a tool for matching tumor genomes to treatment options. In brief, this tool filters actionable somatic variants and delivers the results in evidence-based reports. This filtered list of actionable variants is aimed to assist bioinformaticians and oncologists in their task of interpreting somatic variants prior to MTB discussions. In our analysis, the presented framework was able to identify potentially actionable variants in 94% of cancer patients according to TCGA cohort, and, most importantly, it was able to include experts’ treatment suggestions in a retrospective analysis.

Remarkably, the application of the report strategy on TCGA dataset successfully found actionable variants in 9.9% of patients considering only approved drugs (A1), increasing to 22.7% when considering *off-label* use (B1), and to 89% with evidence from clinical trials (B2). These results were shown to be in line with previous *in silico* efforts to characterize the therapeutic landscape of public datasets [18–20, 35]. Even though these studies slightly differ in the percentages, the overall message remains the same: reporting lower-evidence variants (i.e., *off-label* scenarios and on substances in clinical trials) undeniably increases treatment recommendations for cancer patients. Nevertheless, these results do not mean that 89% of patients can be treated based on a genomic rationale, but rather highlights a trend of increasing opportunities for personalized cancer treatment.

Recent prospective trials using NGS to guide treatment decisions have reported actionable variants in more than 80% of patients (in the most inclusive sense of the word, as used in the current study), or around 50% of patients (attending to a narrower definition, defined as actionable variants for approved drugs and accessible clinical trials) [10–16, 35]. Altogether these studies confirmed the extent of reporting methods; unfortunately, they also showed a gap between identifying actionable alterations and the actual numbers of treated patients (5–13%). The inability to prescribe treatments based on NGS results was in general due to the impossibility to prescribe drugs in off-label scenarios, restricted access to clinical trials, and clinical deterioration. Whereas continuous efforts in molecular characterization of tumor diseases and successful targeted treatment approaches keep raising the expectations on precision medicine, the effect of individual NGS-based treatment decisions on patient outcome is still under discussion in several open clinical trials (SHIVA02, IMPACT, MOSCATO, NCI-MATCH, NCT MASTER).

In the same line, it is important to emphasize that clinical interpretation of somatic variants is subject to many challenges, comprehensively reviewed by [2, 5, 22]. Among them, determining the clinical relevance of a biomarker–drug association is one of the main difficulties. A common approach is to classify the findings into categories or tiers, what we refer to here as “levels of evidence”. Previously proposed classification systems have between three and six levels, and consider informative variables such as validation stage of the drug–biomarker predictive association (FDA approved, clinical trials, case studies, preclinical) [10, 12, 22], histology type [12, 22, 36], gene variant [36], and biological significance [10, 12]. Our six levels of evidence system emphasizes the validation stage of the biomarker–drug predictive association (axis 1–3) because drug availability has been identified as one of the main reasons of the low impact of genomic-driven cancer therapies [10, 11]. In addition, it accounts for histology type (axis A–B) to better inform *off-label* use. That being said, we do not aim to substitute the other classifications, but rather to ease the assessment of the variants in the reports. In a broader perspective, the same is applicable to the main goal of the MTB reports. They aim at reducing the work load that represents searching for actionable variants rather than suggesting just the best treatment option: this is a task for clinicians. We designed our method to include the maximum knowledge important for clinical decisions, but keeping the presentation as simple and compressed as possible. As a consequence, one should be very careful with all recommendations since, although backed by literature, many actionable variants can be far from real clinical practice.

The retrospective analysis established a proof-of-concept of the reports, but also shed light on ways to improve upon the framework. For instance, our reports did not capture indirect targeting unless they were present in the databases, which led to an omission of some experts’ suggestions. For that, we believe it is important to provide the possibility of visualizing somatic alterations in their pathway context. Also, our reports missed target–drug associations such as *LCK*–*dasatinib* because *LCK* mutations have not been shown to predict drug response. This could be improved by including drugs and their targets in the aforementioned pathway visualization. Nevertheless, as we state throughout this article, inferring causality between target gene and drug response is not straightforward. This is the reason why we have so far focused the tool only towards variants with proven evidence of drug response, yet this evidence is not always strong enough and needs to be assessed by a clinician with regard to the clinical course of each patient. For all these reasons, it is important to stress that the reports are aimed at reducing the time-consuming task of linking genomic variants to clinical evidence but they always need a reevaluation by a clinician. Finally, other interesting information that could be included are germline variants. Besides non-cancer syndromes and pharmacodynamic responses, these variants are being demonstrated also to have therapeutic utility in some cases, such as in prostate cancer and childhood leukemias [37, 38].

Taken together, the results of this study have further strengthened the hypothesis that an automated reporting of potential therapeutic options is crucial to make the clinical interpretation of NGS a task with a clinically acceptable turn-around time. We have shown that our method is able to reach a large number of cancer patients. Also, we have demonstrated the reproducibility of experts’ suggestions. This work establishes a proof-of-concept of MTB reports, which provide a structured picture of the actionable variants of a tumor with the already pre-filtered information necessary for deeper treatment discussions.

## Conclusions

We have shown here a method that relies entirely on public knowledge, hence encouraging transparency and joint public efforts for a curated database of actionable variants [3]. We strictly report evidence-based variants, so that the clinician can easily verify with reference the causality between variant and drug response. We encourage the design and enrollment of patients into biomarker-driven clinical trials in order to generate more evidence. Nevertheless, this effort must be accompanied by a systematic recording of treatment decisions and outcomes of patients, also of single case studies [39]. We believe that if NGS is undeniably entering the clinical context, a standardized method to annotate public knowledge of actionable variants is crucial to accomplish a successful and appropriate use of the data.

## Additional files

Additional file 1: List of predictive biomarkers. (XLSX 14 kb)

Additional file 2: Summary of NCT MASTER cohort. (XLSX 334 kb)

Additional file 3: Percentage of patients of the top ten biomarkers at each level of evidence in each tumor type. Level 2 is divided into three further groups: 2a (late clinical trials), 2b (early clinical trials), and 2c (case reports). (PDF 43 kb)

Additional file 4: Heatmap representation of the distribution of identified predictive biomarkers in the GENIE cohort. (EPS 87 kb)

Additional file 5: Eleven reports of NCT MASTER patients. (PDF 175 kb)

Additional file 6: R code of the presented method. The user can generate MTB reports for TCGA samples. (RAR 237 kb)

## Abbreviations

CIViC: Clinical Interpretation of Variants in Cancer database
CNV: Copy number variation
FDA: The Food and Drug Administration
GDKD: Gene Drug Knowledge Database
GENIE: Genomics Evidence Neoplasia Information Exchange
MASTER: Molecularly Aided Stratification for Tumor Eradication Research
MTB: Molecular tumor board
NCCN: National Comprehensive Cancer Network
NCT: Nationale Centrum für Tumorerkrankungen
NGS: Next-generation sequencing
PM: Precision medicine
SNV: Single nucleotide variation
TARGET: Tumor Alterations Relevant for Genomics-driven Therapy Database
TB: Tumor board
TCGA: The Cancer Genome Atlas

## References

[1] Center for Drug Evaluation and Research. Genomics - Table of Pharmacogenomic Biomarkers in Drug Labeling. https://www.fda.gov/Drugs/ScienceResearch/ResearchAreas/Pharmacogenetics/ucm083378.htm. Accessed 6 Mar 2017.

[2] Vidwans SJ, Turski ML, Janku F, Garrido-Laguna I, Munoz J, Schwab R (2014). A framework for genomic biomarker actionability and its use in clinical decision making. Oncoscience.

[3] Good BM, Ainscough BJ, McMichael JF, Su AI, Griffith OL (2014). Organizing knowledge to enable personalization of medicine in cancer. Genome Biol.

[4] Dienstmann R, Dong F, Borger D, Dias-Santagata D, Ellisen LW, Le LP (2014). Standardized decision support in next generation sequencing reports of somatic cancer variants. Mol Oncol.

[5] Johnson A, Zeng J, Bailey AM, Holla V, Litzenburger B, Lara-Guerra H (2015). The right drugs at the right time for the right patient: the MD Anderson precision oncology decision support platform. Drug Discov Today.

[6] Welch BM, Kawamoto K (2013). The need for clinical decision support integrated with the electronic health record for the clinical application of whole genome sequencing information. J Pers Med.

[7] Tourneau CL, Kamal M, Tsimberidou A-M, Bedard P, Pierron G, Callens C (2016). Treatment algorithms based on tumor molecular profiling: the essence of precision medicine trials. J Natl Cancer Inst.

[8] André F, Bachelot T, Commo F, Campone M, Arnedos M, Dieras V (2014). Comparative genomic hybridisation array and DNA sequencing to direct treatment of metastatic breast cancer: a multicentre, prospective trial (SAFIR01/UNICANCER). Lancet Oncol.

[9] Roychowdhury S, Iyer MK, Robinson DR, Lonigro RJ, Wu Y-M, Cao X (2011). Personalized oncology through integrative high-throughput sequencing: a pilot study. Sci Transl Med.

[10] Beltran H, Eng K, Mosquera JM, Sigaras A, Romanel A, Rennert H (2015). Whole-exome sequencing of metastatic cancer and biomarkers of treatment response. JAMA Oncol.

[11] Sohal DPS, Rini BI, Khorana AA, Dreicer R, Abraham J, Procop GW (2016). Prospective clinical study of precision oncology in solid tumors. J Natl Cancer Inst.

[12] Van Allen EM, Wagle N, Stojanov P, Perrin DL, Cibulskis K, Marlow S (2014). Whole-exome sequencing and clinical interpretation of formalin-fixed, paraffin-embedded tumor samples to guide precision cancer medicine. Nat Med.

[13] Le Tourneau C, Delord J-P, Gonçalves A, Gavoille C, Dubot C, Isambert N (2015). Molecularly targeted therapy based on tumour molecular profiling versus conventional therapy for advanced cancer (SHIVA): a multicentre, open-label, proof-of-concept, randomised, controlled phase 2 trial. Lancet Oncol.

[14] Rennert H, Eng K, Zhang T, Tan A, Xiang J, Romanel A (2016). Development and validation of a whole-exome sequencing test for simultaneous detection of point mutations, indels and copy-number alterations for precision cancer care. NPJ Genomic Med.

[15] Tsimberidou A-M, Iskander NG, Hong DS, Wheler JJ, Falchook GS, Fu S (2012). Personalized medicine in a phase I clinical trials program: the MD Anderson Cancer Center Initiative. Clin Cancer Res.

[16] Tsimberidou A-M, Wen S, Hong DS, Wheler JJ, Falchook GS, Fu S (2014). Personalized medicine for patients with advanced cancer in the phase I program at MD Anderson: validation and landmark analyses. Clin Cancer Res.

[17] Roychowdhury S, Chinnaiyan AM (2016). Translating cancer genomes and transcriptomes for precision oncology. CA Cancer J Clin.

[18] Chakravarty D, Gao J, Phillips S, Kundra R, Zhang H, Wang J, et al. OncoKB: a precision oncology knowledge base. JCO Precis Oncol. 2017;1:1–16. 10.1200/PO.17.00011 PMC5586540 28890946

[19] Rubio-Perez C, Tamborero D, Schroeder MP, Antolín AA, Deu-Pons J, Perez-Llamas C (2015). In silico prescription of anticancer drugs to cohorts of 28 tumor types reveals targeting opportunities. Cancer Cell.

[20] Dienstmann R, Jang IS, Bot B, Friend S, Guinney J (2015). Database of genomic biomarkers for cancer drugs and clinical targetability in solid tumors. Cancer Discov.

[21] Griffith M, Spies NC, Krysiak K, McMichael JF, Coffman AC, Danos AM (2017). CIViC is a community knowledgebase for expert crowdsourcing the clinical interpretation of variants in cancer. Nat Genet.

[22] Meric-Bernstam F, Johnson A, Holla V, Bailey AM, Brusco L, Chen K (2015). A decision support framework for genomically informed investigational cancer therapy. J Natl Cancer Inst.

[23] The Cancer Genome Atlas Network (2012). Comprehensive molecular portraits of human breast tumours. Nature.

[24] Consortium TAPG (2017). AACR project GENIE: powering precision medicine through an international consortium. Cancer Discov.

[25] Li H, Durbin R (2009). Fast and accurate short read alignment with Burrows–Wheeler transform. Bioinformatics.

[26] Li H, Handsaker B, Wysoker A, Fennell T, Ruan J, Homer N (2009). The Sequence Alignment/Map format and SAMtools. Bioinformatics.

[27] Yaktapour N, Meiss F, Mastroianni J, Zenz T, Andrlova H, Mathew NR (2014). BRAF inhibitor–associated ERK activation drives development of chronic lymphocytic leukemia. J Clin Invest.

[28] Jones DTW, Jäger N, Kool M, Zichner T, Hutter B, Sultan M (2012). Dissecting the genomic complexity underlying medulloblastoma. Nature.

[29] Jones DTW, Hutter B, Jäger N, Korshunov A, Kool M, Warnatz H-J (2013). Recurrent somatic alterations of FGFR1 and NTRK2 in pilocytic astrocytoma. Nat Genet.

[30] Rimmer A, Phan H, Mathieson I, Iqbal Z, Twigg SRF, Wgs500 Consortium, et al. Integrating mapping-, assembly- and haplotype-based approaches for calling variants in clinical sequencing applications. Nat Genet. 2014;46:912–8. 10.1038/ng.3036 PMC4753679 25017105

[31] Wang K, Li M, Hakonarson H (2010). ANNOVAR: functional annotation of genetic variants from high-throughput sequencing data. Nucleic Acids Res.

[32] Koboldt DC, Zhang Q, Larson DE, Shen D, McLellan MD, Lin L (2012). VarScan 2: Somatic mutation and copy number alteration discovery in cancer by exome sequencing. Genome Res.

[33] Quinlan AR, Hall IM (2010). BEDTools: a flexible suite of utilities for comparing genomic features. Bioinformatics.

[34] Wang J, Mullighan CG, Easton J, Roberts S, Heatley SL, Ma J (2011). CREST maps somatic structural variation in cancer genomes with base-pair resolution. Nat Methods.

[35] Zehir A, Benayed R, Shah RH, Syed A, Middha S, Kim HR (2017). Mutational landscape of metastatic cancer revealed from prospective clinical sequencing of 10,000 patients. Nat Med.

[36] Sukhai MA, Craddock KJ, Thomas M, Hansen AR, Zhang T, Siu L (2016). A classification system for clinical relevance of somatic variants identified in molecular profiling of cancer. Genet Med.

[37] Robinson D, Van Allen EM, Wu Y-M, Schultz N, Lonigro RJ, Mosquera J-M (2015). Integrative clinical genomics of advanced prostate cancer. Cell.

[38] Treviño LR, Yang W, French D, Hunger SP, Carroll WL, Devidas M (2009). Germline genomic variants associated with childhood acute lymphoblastic leukemia. Nat Genet.

[39] Rubin MA (2015). Make precision medicine work for cancer care. Nature.

