# Supplementary material for: From somatic variants towards precision oncology: Evidence-driven reporting of treatment options in molecular tumor boards
Source: Genome Med. 2018 Mar 15;10:18. doi: 10.1186/s13073-018-0529-2 (PMC5856211; doi:10.1186/s13073-018-0529-2)
Supplement: Supplementary file 3 — Percentage of patients of the top ten biomarkers at each level of evidence in each tumor type. Level 2 is divided into three further groups: 2a (late clinical trials), 2b (early clinical trials), and 2c (case reports). (PDF 43 kb) [file 13073_2018_529_MOESM3_ESM.pdf]

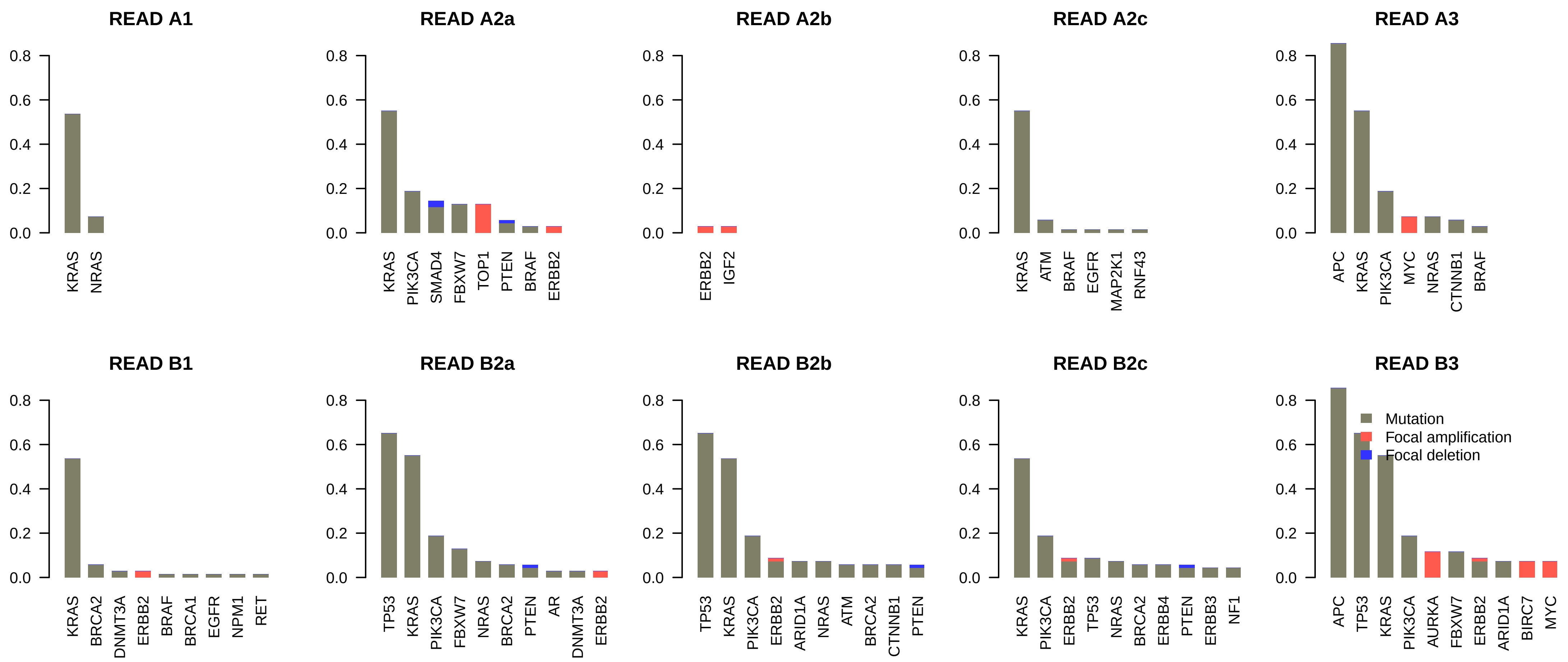
READ A1
KRAS
NRAS
READ A2a
KRAS
PIK3CA
SMAD4
FBXW7
TOP1
PTEN
BRAF
ERBB2
READ A2b
ERBB2
IGF2
READ A2c
KRAS
ATM
BRAF
EGFR
MAP2K1
RNF43
READ A3
APC
KRAS
PIK3CA
MYC
NRAS
CTNNB1
BRAF
READ B1
KRAS
BRCA2
DNMT3A
ERBB2
BRAF
BRCA1
EGFR
NPM1
RET
READ B2a
TP53
KRAS
PIK3CA
FBXW7
NRAS
BRCA2
PTEN
AR
DNMT3A
ERBB2
READ B2b
TP53
KRAS
PIK3CA
ERBB2
ARID1A
NRAS
ATM
BRCA2
CTNNB1
PTEN
READ B2c
KRAS
PIK3CA
ERBB2
TP53
NRAS
BRCA2
ERBB4
PTEN
ERBB3
NF1
READ B3
APC
TP53
KRAS
PIK3CA
AURKA
FBXW7
ERBB2
ARID1A
BIRC7
MYC
Mutation
Focal amplification
Focal deletion
0.0
0.2
0.4
0.6
0.8

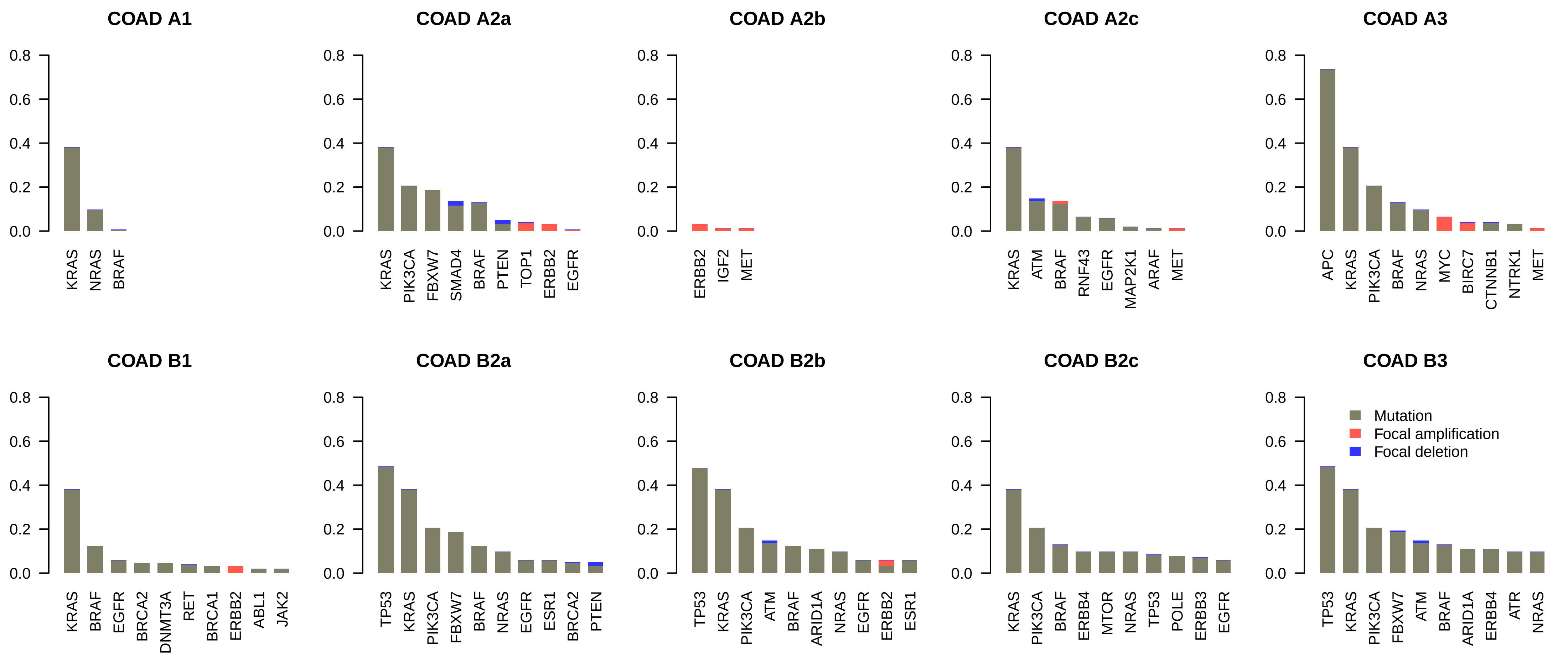
COAD A1
KRAS
NRAS
BRAF
COAD A2a
KRAS
PIK3CA
FBXW7
SMAD4
BRAF
PTEN
TOP1
ERBB2
EGFR
COAD A2b
ERBB2
IGF2
MET
COAD A2c
KRAS
ATM
BRAF
RNF43
EGFR
MAP2K1
ARAF
MET
COAD A3
APC
KRAS
PIK3CA
BRAF
NRAS
MYC
BIRC7
CTNNB1
NTRK1
MET
COAD B1
KRAS
BRAF
EGFR
BRCA2
DNMT3A
RET
BRCA1
ERBB2
ABL1
JAK2
COAD B2a
TP53
KRAS
PIK3CA
FBXW7
BRAF
NRAS
EGFR
ESR1
BRCA2
PTEN
COAD B2b
TP53
KRAS
PIK3CA
ATM
BRAF
ARID1A
NRAS
EGFR
ERBB2
ESR1
COAD B2c
KRAS
PIK3CA
BRAF
ERBB4
MTOR
NRAS
TP53
POLE
ERBB3
EGFR
COAD B3
TP53
KRAS
PIK3CA
FBXW7
ATM
BRAF
ARID1A
ERBB4
ATR
NRAS
Mutation
Focal amplification
Focal deletion
0.0
0.2
0.4
0.6
0.8

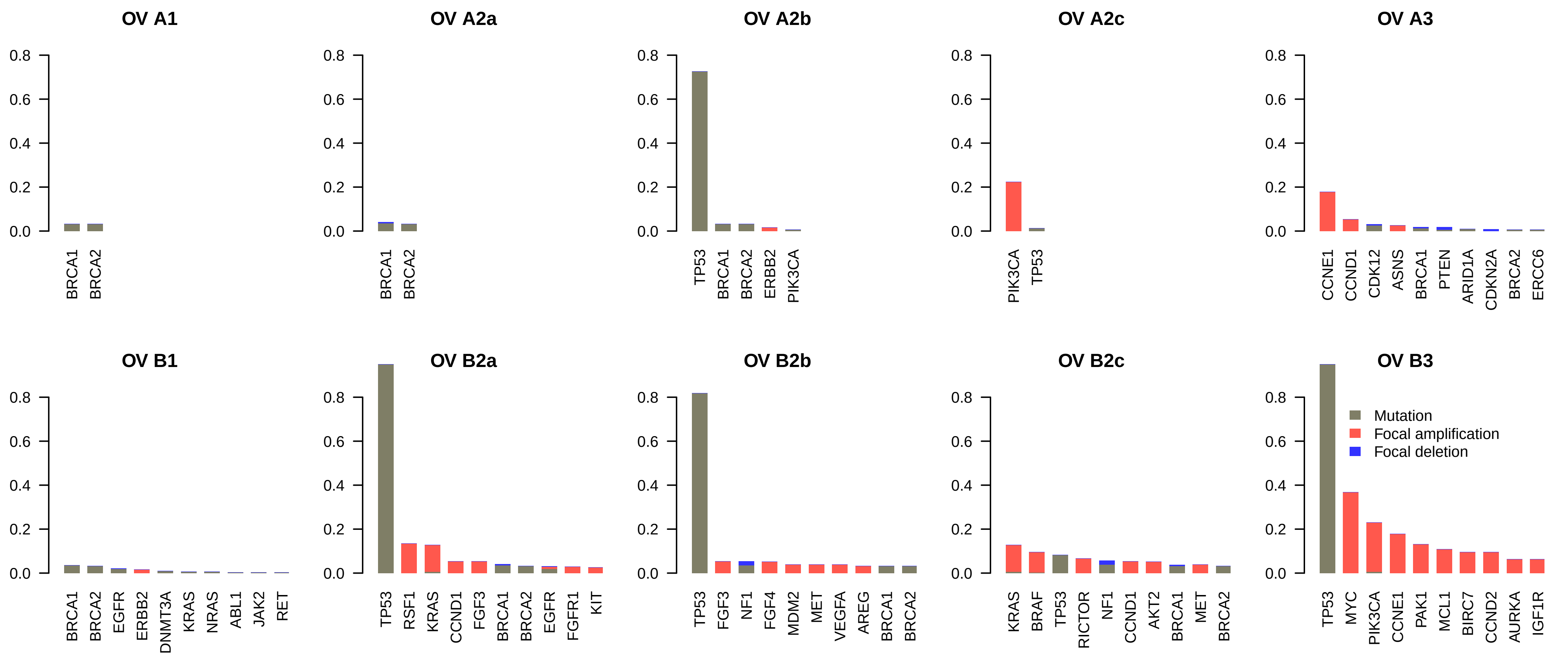

OV A1
OV A2a
OV A2b
OV A2c
OV A3
OV B1
OV B2a
OV B2b
OV B2c
OV B3
0.0
0.2
0.4
0.6
0.8
BRCA1 BRCA2
BRCA1 BRCA2
TP53 BRCA1 BRCA2 ERBB2 PIK3CA
PIK3CA TP53
CCNE1 CCND1 CDK12 ASNS BRCA1 PTEN ARID1A CDKN2A BRCA2 ERCC6
BRCA1 BRCA2 EGFR ERBB2 DNMT3A KRAS NRAS ABL1 JAK2 RET
TP53 RSF1 KRAS CCND1 FGF3 BRCA1 BRCA2 EGFR FGFR1 KIT
TP53 FGF3 NF1 FGF4 MDM2 MET VEGFA AREG BRCA1 BRCA2
KRAS BRAF TP53 RICTOR NF1 CCND1 AKT2 BRCA1 MET BRCA2
TP53 MYC PIK3CA CCNE1 PAK1 MCL1 BIRC7 CCND2 AURKA IGF1R
Mutation
Focal amplification
Focal deletion

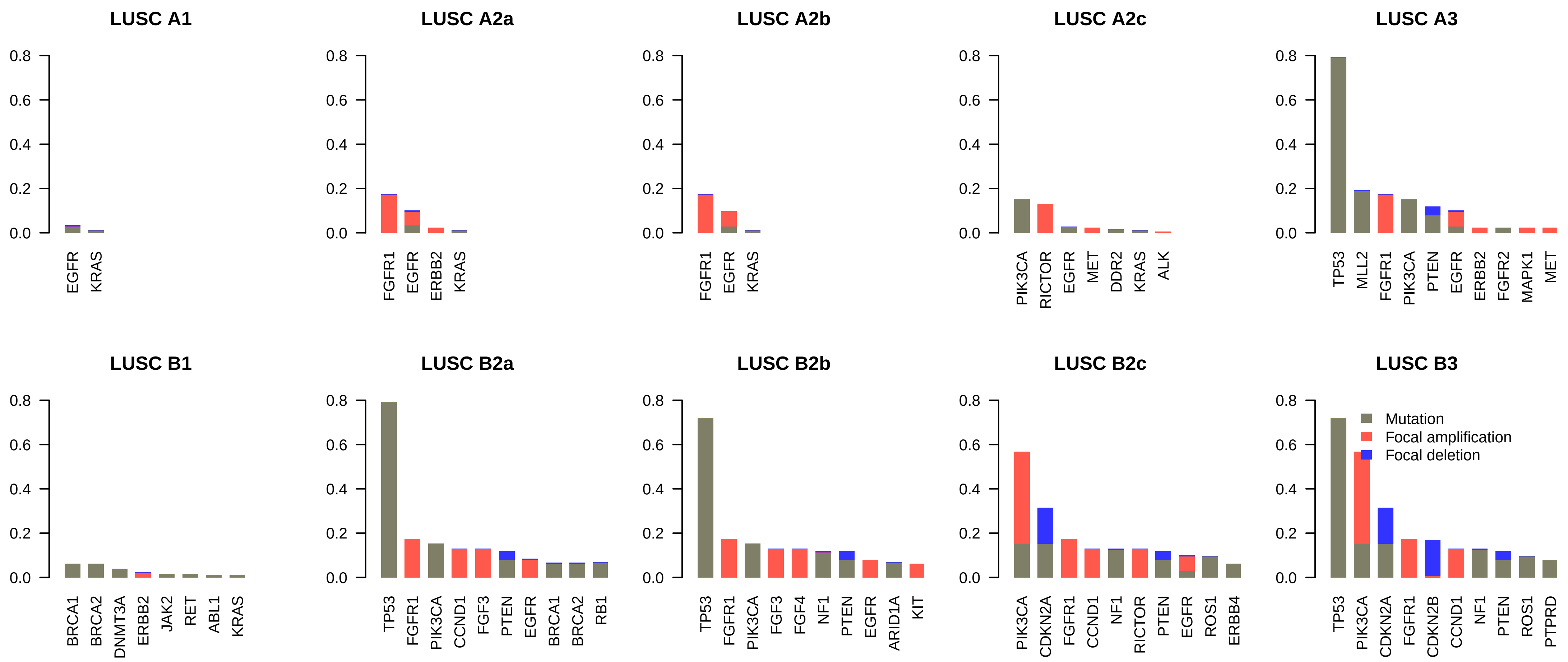
LUSC A1
EGFR
KRAS
LUSC A2a
FGFR1
EGFR
ERBB2
KRAS
LUSC A2b
FGFR1
EGFR
KRAS
LUSC A2c
PIK3CA
RICTOR
EGFR
MET
DDR2
KRAS
ALK
LUSC A3
TP53
MLL2
FGFR1
PIK3CA
PTEN
EGFR
ERBB2
FGFR2
MAPK1
MET
LUSC B1
BRCA1
BRCA2
DNMT3A
ERBB2
JAK2
RET
ABL1
KRAS
LUSC B2a
TP53
FGFR1
PIK3CA
CCND1
FGF3
PTEN
EGFR
BRCA1
BRCA2
RB1
LUSC B2b
TP53
FGFR1
PIK3CA
FGF3
FGF4
NF1
PTEN
EGFR
ARID1A
KIT
LUSC B2c
PIK3CA
CDKN2A
FGFR1
CCND1
NF1
RICTOR
PTEN
EGFR
ROS1
ERBB4
LUSC B3
TP53
PIK3CA
CDKN2A
FGFR1
CDKN2B
CCND1
NF1
PTEN
ROS1
PTPRD
Mutation
Focal amplification
Focal deletion
0.0
0.2
0.4
0.6
0.8

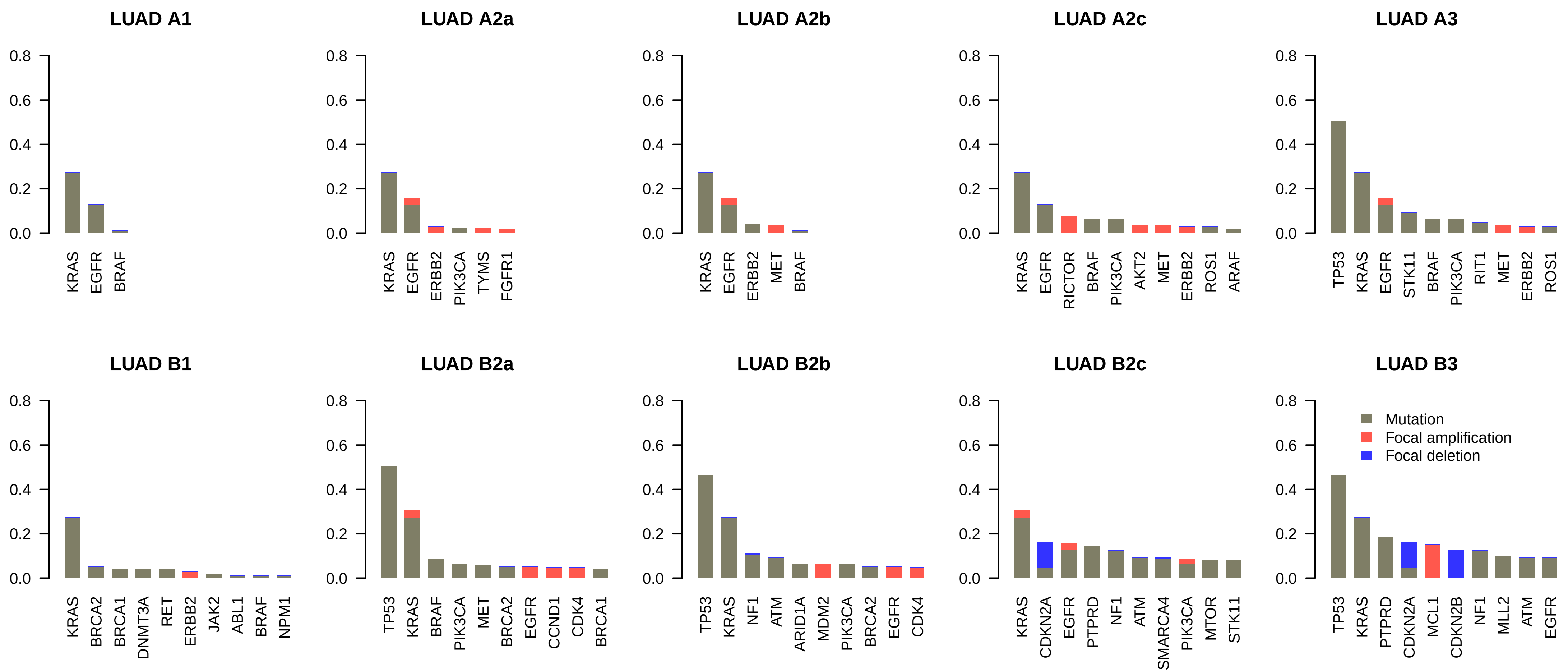
LUAD A1
KRAS
EGFR
BRAF
LUAD A2a
KRAS
EGFR
ERBB2
PIK3CA
TYMS
FGFR1
LUAD A2b
KRAS
EGFR
ERBB2
MET
BRAF
LUAD A2c
KRAS
EGFR
RICTOR
BRAF
PIK3CA
AKT2
MET
ERBB2
ROS1
ARAF
LUAD A3
TP53
KRAS
EGFR
STK11
BRAF
PIK3CA
RIT1
MET
ERBB2
ROS1
LUAD B1
KRAS
BRCA2
BRCA1
DNMT3A
RET
ERBB2
JAK2
ABL1
BRAF
NPM1
LUAD B2a
TP53
KRAS
BRAF
PIK3CA
MET
BRCA2
EGFR
CCND1
CDK4
BRCA1
LUAD B2b
TP53
KRAS
NF1
ATM
ARID1A
MDM2
PIK3CA
BRCA2
EGFR
CDK4
LUAD B2c
KRAS
CDKN2A
EGFR
PTPRD
NF1
ATM
SMARCA4
PIK3CA
MTOR
STK11
LUAD B3
TP53
KRAS
PTPRD
CDKN2A
MCL1
CDKN2B
NF1
MLL2
ATM
EGFR
Mutation
Focal amplification
Focal deletion
0.0
0.2
0.4
0.6
0.8

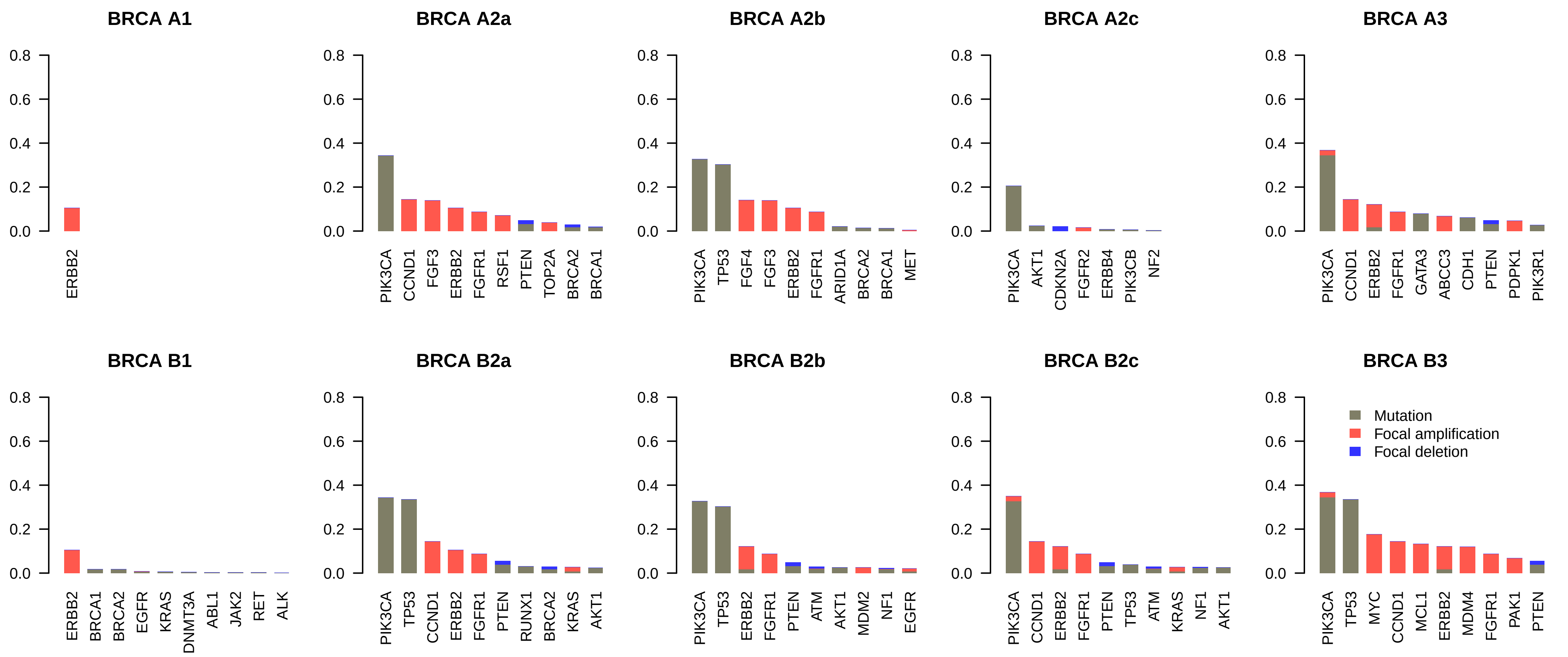

BRCA A1
ERBB2
BRCA A2a
PIK3CA
CCND1
FGF3
ERBB2
FGFR1
RSF1
PTEN
TOP2A
BRCA2
BRCA1
BRCA A2b
PIK3CA
TP53
FGF4
FGF3
ERBB2
FGFR1
ARID1A
BRCA2
BRCA1
MET
BRCA A2c
PIK3CA
AKT1
CDKN2A
FGFR2
ERBB4
PIK3CB
NF2
BRCA A3
PIK3CA
CCND1
ERBB2
FGFR1
GATA3
ABCC3
CDH1
PTEN
PDPK1
PIK3R1
BRCA B1
ERBB2
BRCA1
BRCA2
EGFR
KRAS
DNMT3A
ABL1
JAK2
RET
ALK
BRCA B2a
PIK3CA
TP53
CCND1
ERBB2
FGFR1
PTEN
RUNX1
BRCA2
KRAS
AKT1
BRCA B2b
PIK3CA
TP53
ERBB2
FGFR1
PTEN
ATM
AKT1
MDM2
NF1
EGFR
BRCA B2c
PIK3CA
CCND1
ERBB2
FGFR1
PTEN
TP53
ATM
KRAS
NF1
AKT1
BRCA B3
PIK3CA
TP53
MYC
CCND1
MCL1
ERBB2
MDM4
FGFR1
PAK1
PTEN
0.0
0.2
0.4
0.6
0.8
Mutation
Focal amplification
Focal deletion

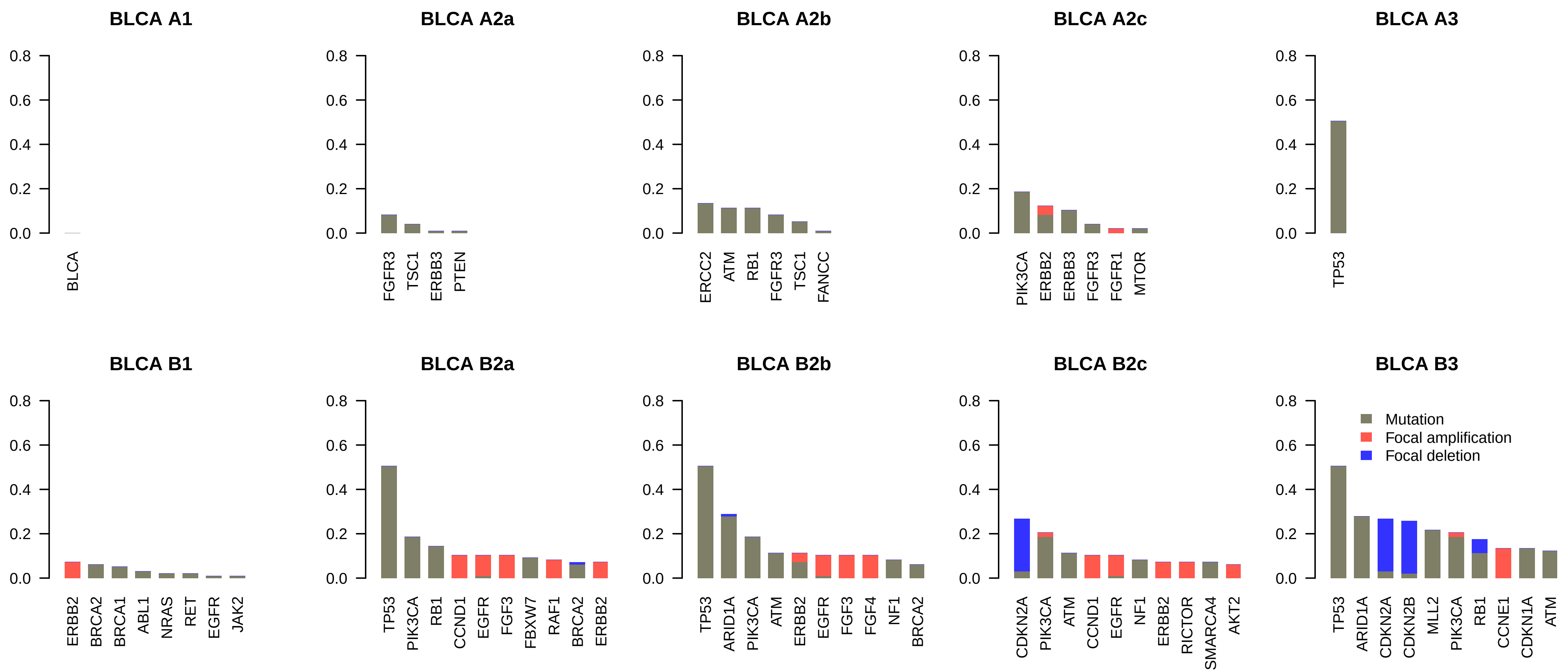
BLCA A1
BLCA A2a
BLCA A2b
BLCA A2c
BLCA A3
BLCA B1
BLCA B2a
BLCA B2b
BLCA B2c
BLCA B3
0.0
0.2
0.4
0.6
0.8
BLCA
FGFR3
TSC1
ERBB3
PTEN
ERCC2
ATM
RB1
FGFR3
TSC1
FANCC
PIK3CA
ERBB2
ERBB3
FGFR3
FGFR1
MTOR
TP53
ERBB2
BRCA2
BRCA1
ABL1
NRAS
RET
EGFR
JAK2
TP53
PIK3CA
RB1
CCND1
EGFR
FGF3
FBXW7
RAF1
BRCA2
ERBB2
TP53
ARID1A
PIK3CA
ATM
ERBB2
EGFR
FGF3
FGF4
NF1
BRCA2
CDKN2A
PIK3CA
ATM
CCND1
EGFR
NF1
ERBB2
RICTOR
SMARCA4
AKT2
TP53
ARID1A
CDKN2A
CDKN2B
MLL2
PIK3CA
RB1
CCNE1
CDKN1A
ATM
Mutation
Focal amplification
Focal deletion

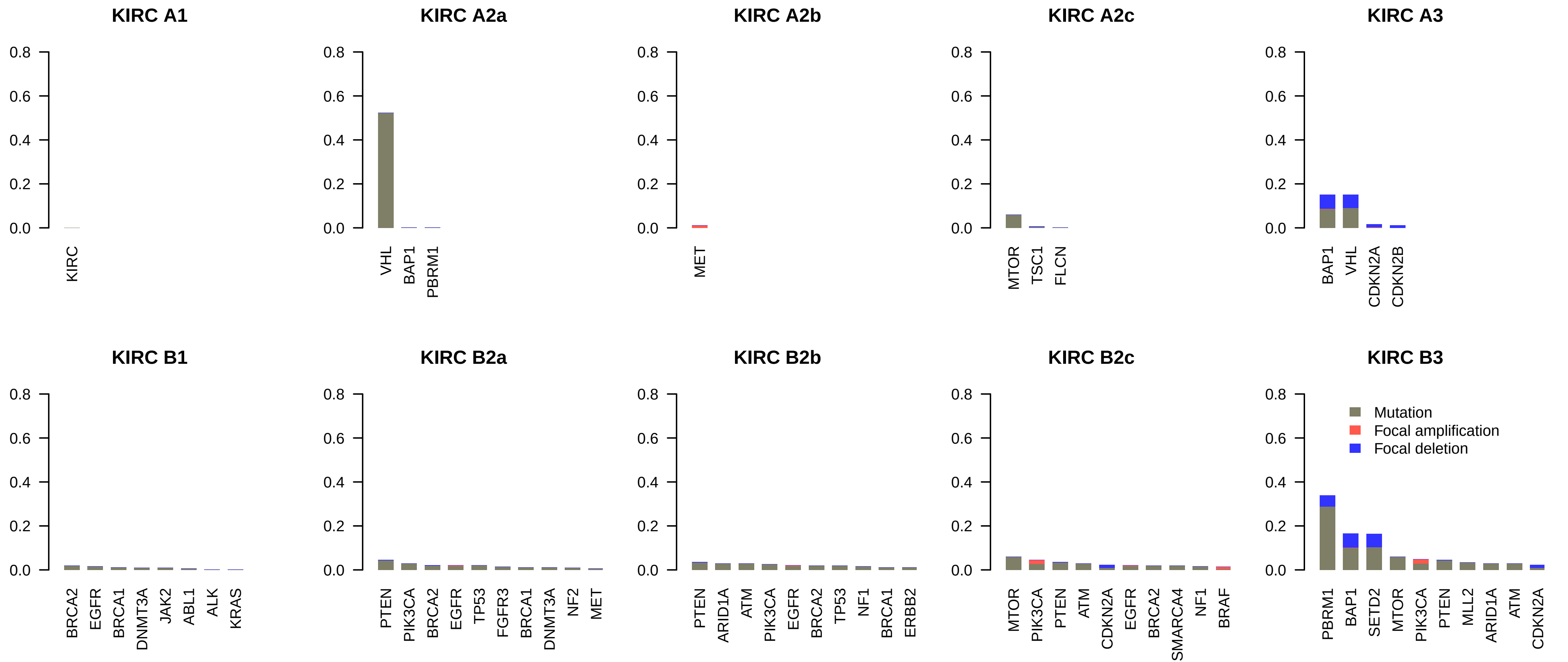

KIRC A1
KIRC A2a
KIRC A2b
KIRC A2c
KIRC A3
0.8
0.6
0.4
0.2
0.0
KIRC
VHL
BAP1
PBRM1
MET
MTOR
TSC1
FLCN
BAP1
VHL
CDKN2A
CDKN2B
KIRC B1
KIRC B2a
KIRC B2b
KIRC B2c
KIRC B3
BRCA2
EGFR
BRCA1
DNMT3A
JAK2
ABL1
ALK
KRAS
PTEN
PIK3CA
BRCA2
EGFR
TP53
FGFR3
BRCA1
DNMT3A
NF2
MET
PTEN
ARID1A
ATM
PIK3CA
EGFR
BRCA2
TP53
NF1
BRCA1
ERBB2
MTOR
PIK3CA
PTEN
ATM
CDKN2A
EGFR
BRCA2
SMARCA4
NF1
BRAF
PBRM1
BAP1
SETD2
MTOR
PIK3CA
PTEN
MLL2
ARID1A
ATM
CDKN2A
Mutation
Focal amplification
Focal deletion

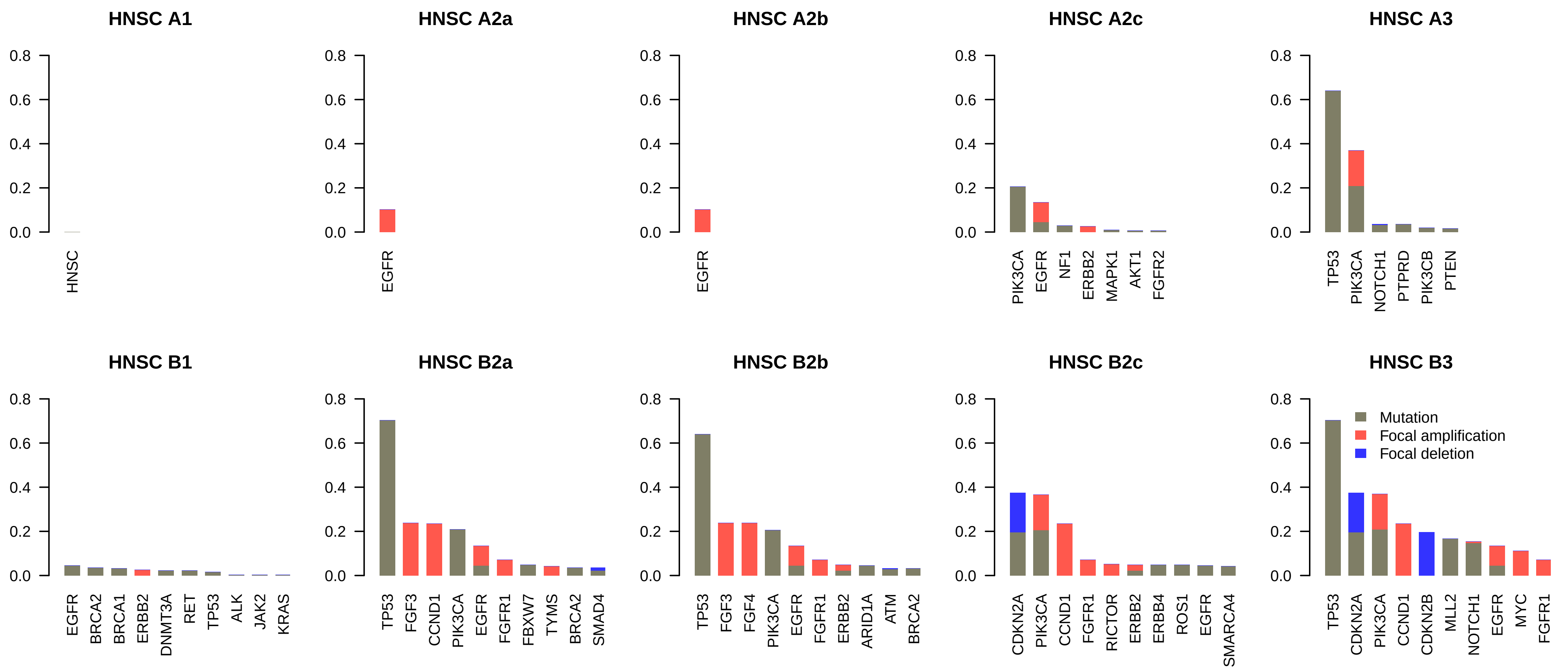

HNSC A1
HNSC A2a
HNSC A2b
HNSC A2c
HNSC A3
HNSC B1
HNSC B2a
HNSC B2b
HNSC B2c
HNSC B3
0.8
0.6
0.4
0.2
0.0
HNSC
EGFR
EGFR
PIK3CA EGFR NF1 ERBB2 MAPK1 AKT1 FGFR2
TP53 PIK3CA NOTCH1 PTPRD PIK3CB PTEN
EGFR BRCA2 BRCA1 ERBB2 DNMT3A RET TP53 ALK JAK2 KRAS
TP53 FGF3 CCND1 PIK3CA EGFR FGFR1 FBXW7 TYMS BRCA2 SMAD4
TP53 FGF3 FGF4 PIK3CA EGFR FGFR1 ERBB2 ARID1A ATM BRCA2
CDKN2A PIK3CA CCND1 FGFR1 RICTOR ERBB2 ERBB4 ROS1 EGFR SMARCA4
TP53 CDKN2A PIK3CA CCND1 CDKN2B MLL2 NOTCH1 EGFR MYC FGFR1
Mutation
Focal amplification
Focal deletion

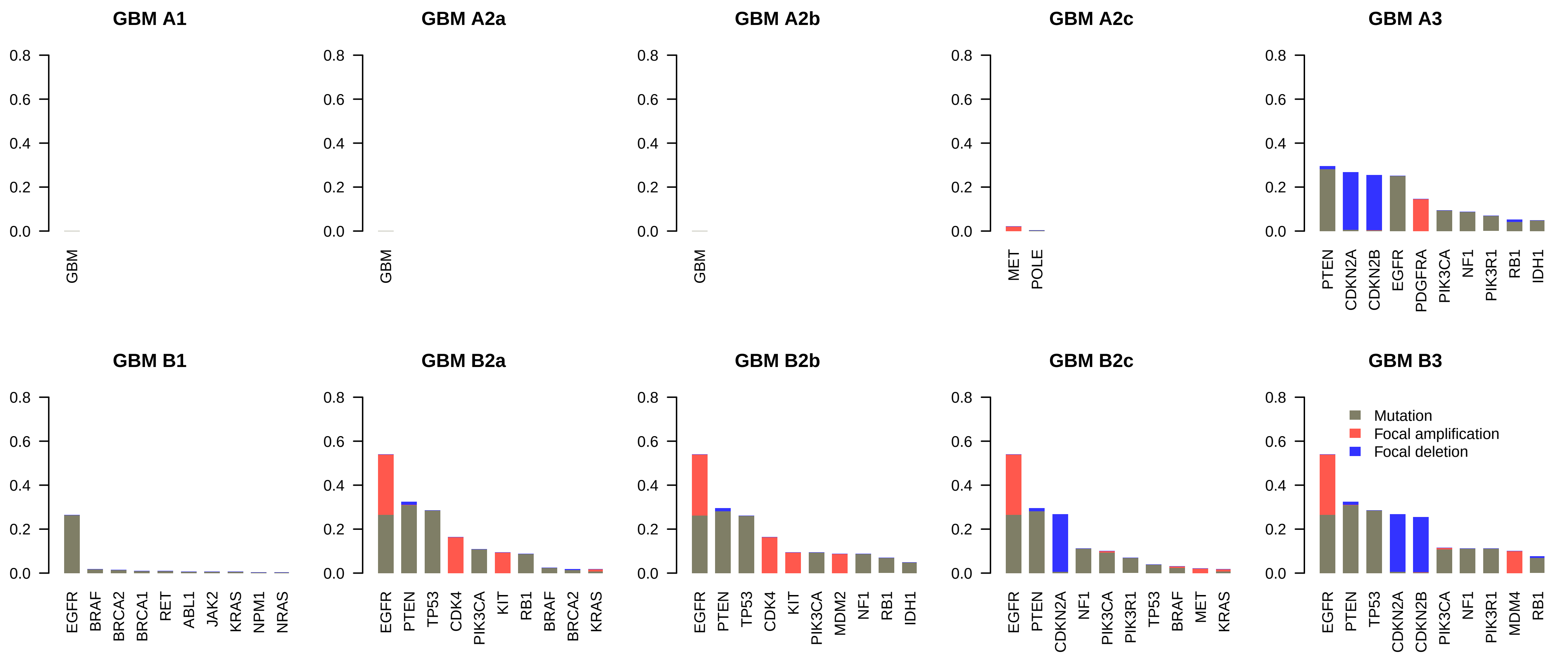
GBM A1
GBM A2a
GBM A2b
GBM A2c
GBM A3
0.8
0.6
0.4
0.2
0.0
GBM
MET
POLE
PTEN
CDKN2A
CDKN2B
EGFR
PDGFRA
PIK3CA
NF1
PIK3R1
RB1
IDH1
GBM B1
GBM B2a
GBM B2b
GBM B2c
GBM B3
EGFR
BRAF
BRCA2
BRCA1
RET
ABL1
JAK2
KRAS
NPM1
NRAS
PTEN
TP53
CDK4
PIK3CA
KIT
RB1
BRAF
BRCA2
KRAS
MDM2
NF1
IDH1
CDKN2A
PIK3R1
MET
CDKN2B
MDM4
Mutation
Focal amplification
Focal deletion

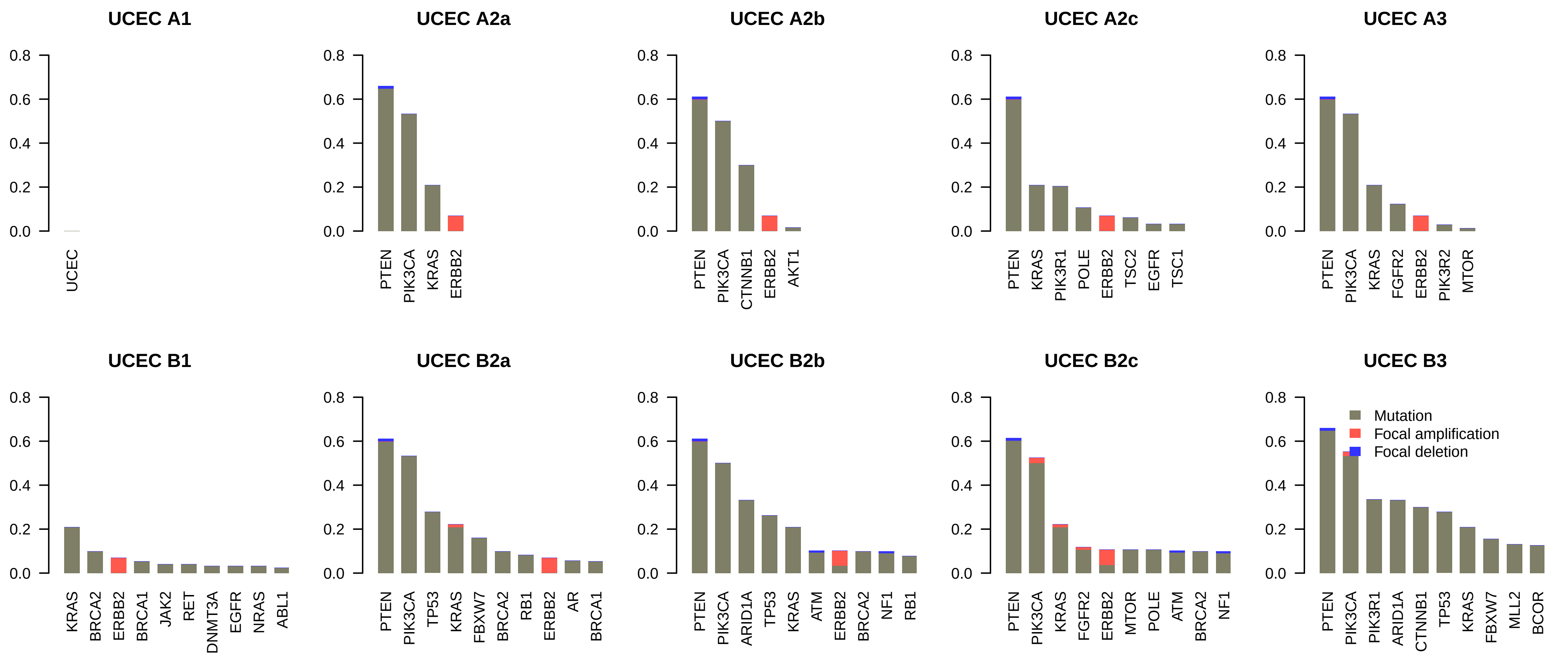

UCEC A1
UCEC A2a
UCEC A2b
UCEC A2c
UCEC A3
UCEC B1
UCEC B2a
UCEC B2b
UCEC B2c
UCEC B3
0.8
0.6
0.4
0.2
0.0
UCEC
PTEN PIK3CA KRAS ERBB2
PTEN PIK3CA CTNNB1 ERBB2 AKT1
PTEN KRAS PIK3R1 POLE ERBB2 TSC2 EGFR TSC1
PTEN PIK3CA KRAS FGFR2 ERBB2 PIK3R2 MTOR
KRAS BRCA2 ERBB2 BRCA1 JAK2 RET DNMT3A EGFR NRAS ABL1
PTEN PIK3CA TP53 KRAS FBXW7 BRCA2 RB1 ERBB2 AR BRCA1
PTEN PIK3CA ARID1A TP53 KRAS ATM ERBB2 BRCA2 NF1 RB1
PTEN PIK3CA KRAS FGFR2 ERBB2 MTOR POLE ATM BRCA2 NF1
PTEN PIK3CA PIK3R1 ARID1A CTNNB1 TP53 KRAS FBXW7 MLL2 BCOR
Mutation
Focal amplification
Focal deletion

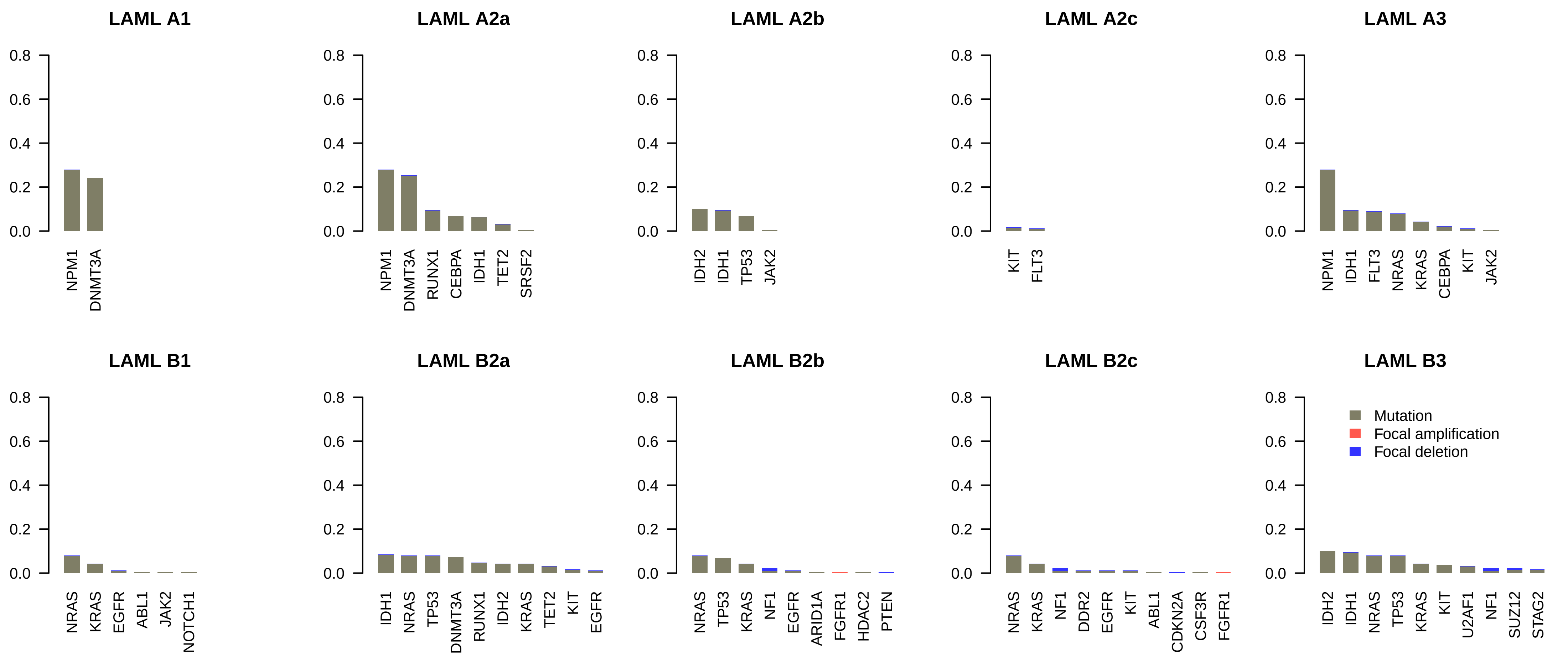

LAML A1
LAML A2a
LAML A2b
LAML A2c
LAML A3
LAML B1
LAML B2a
LAML B2b
LAML B2c
LAML B3
0.0
0.2
0.4
0.6
0.8
NPM1 DNMT3A
NPM1 DNMT3A RUNX1 CEBPA IDH1 TET2 SRSF2
IDH2 IDH1 TP53 JAK2
KIT FLT3
NPM1 IDH1 FLT3 NRAS KRAS CEBPA KIT JAK2
NRAS KRAS EGFR ABL1 JAK2 NOTCH1
IDH1 NRAS TP53 DNMT3A RUNX1 IDH2 KRAS TET2 KIT EGFR
NRAS TP53 KRAS NF1 EGFR ARID1A FGFR1 HDAC2 PTEN
NRAS KRAS NF1 DDR2 EGFR KIT ABL1 CDKN2A CSF3R FGFR1
IDH2 IDH1 NRAS TP53 KRAS KIT U2AF1 NF1 SUZ12 STAG2
Mutation
Focal amplification
Focal deletion
