# Supplementary material for: From somatic variants towards precision oncology: Evidence-driven reporting of treatment options in molecular tumor boards
Source: Genome Med. 2018 Mar 15;10:18. doi: 10.1186/s13073-018-0529-2 (PMC5856211; doi:10.1186/s13073-018-0529-2)
Supplement: Supplementary file 5 — Eleven reports of NCT MASTER patients. (PDF 175 kb) [file 13073_2018_529_MOESM5_ESM.pdf]

MTB Report - From somatic variants to treatment options

Department of Medical Statistics, University Medical Center Goettingen

September 27, 2017

PATIENT INFORMATION

**Patient ID**	MASTER-01	**Tissue Type**	?
**Gender**	Male	**Tumor Content (%)**	35
**Disease**	Breast carcinoma metastasis	**Number SNVs**	104
**Previous Therapies**	-	**Number CNVs**	3045
**Tumor Board Decision**	BRCA1/2 deletions: PARP-Inhibitors (Olaparib, Melfalon); RAF1 and PDGFRA amplifications: sorafenib	**Number Fusions**	5

GENE-DRUG PREDICTIVE ASSOCIATIONS

Method: Somatic variants of the patient (mutations, amplifications, deletions, rearrangments) are searched in curated databases of predictive biomarkers (GKDB[1], CIViC[2]) and reported according to their clinical evidence (see Levels of Evidence). In the following two tables (SNVs and CNVs), basic information of the somatic variants with relevant clinical implications can be found:

Gene	Patient's Variant	Level of Evidence	Variant Freq.	Zygosity	Quality (Phred)
TP53	I162N	A2 ,B2 ,B3	0.42	het	225

Gene	Patient's Variant	Level of Evidence	Seg. Mean	Size (Mb)
AURKA	ampl.	B3	0.55	13.9
BIRC7	ampl.	B3	0.55	13.9
BRCA2	del.	A2 ,B2 ,B3	-1.17	0.1
KIT	ampl.	B2	0.55	18.3
MCL1	ampl.	B3	0.52	31.4
MDM4	ampl.	B3	0.50	23.3
MYC	ampl.	B3	0.67	16.4
PDGFRA	ampl.	B3	0.55	18.3
RAF1	ampl.	B2	0.56	16.0
RB1	del.	A3 ,B3	-0.56	0.3
TOP1	ampl.	B2	0.69	14.7

Levels of Evidence: Findings are classified into 6 levels of evidence combining the **axis A-B** and the **axis 1-2-3**. Level A means evidence in the same cancer type. Level B means evidence in any other cancer type. On the 1-2-3 axis, level 1 means evidence supported by drug approval organizations or clinical guidelines, level 2 contains clinical evidence (clinical trials, case reports) and level 3 consists of preclinical evidence. The distribution of findings into levels is summarized in the right figure

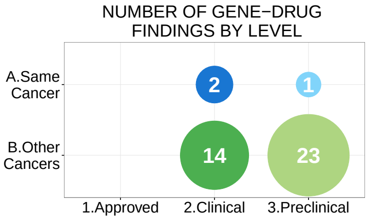

Table of Results: All the predicitve associations are detailed in this table. The results are sorted by 1) drug frequency, 2) levels of evidence (A1-B1-A2-B2-A3-B3). To allow a quick interpretation, the type of association (response, resistance) is colored (green, red) and new variants are gray and underlined.

Patient		Gene-Drug Associations						
**Gene**	**Variant**	**Disease**	**Known Variant**	**Association**	**Drugs**	**Evidence**	**PMID**	**Level**
AURKA	ampl.	unspecified	ampl. (GoF)	sensitivity	AURK inhibitors	preclinical	22302096	B3
		prostate	ampl. (GoF)	sensitivity	AURK inhibitors	preclinical	22389870	B3
MYC	ampl.	myeloma	ampl. (GoF)	sensitivity	BET inhibitors	preclinical	21889194	B3
		neuroblastoma	ampl. (GoF)	sensitivity	BET inhibitors	preclinical	23430699	B3
RAF1	ampl.	melanoma	ampl.	sensitivity	Paclitaxel,Sorafenib,Carboplatin	clin. trials	26307133	B2
TP53	I162N	ovarian	any (GoF)	response	WEE1 inhibitors + carboplatin	early trials	ASCO2015 (abstr 2507)	B2
			mut. (LoF)	resistance	cisplatin	early trials	27646943	B2
BIRC7	ampl.	colorectal	ampl.	resistance or non-response	Cisplatin	preclinical	23188704	B3
TP53	I162N	AML	mut. (LoF)	response	decitabine	early trials	27959731	B2
		MDS	mut. (LoF)	response	decitabine	early trials	27959731	B2
		lung	mut.	resistance or non-response	Docetaxel	preclinical	22425996	B3
		lung	mut.	sensitivity	Docetaxel,Selumetinib (AZD6244)	preclinical	22425996	B3
KIT	ampl.	melanoma	ampl.	resistance or non-response	Imatinib	clin. trials	23775962	B2

[1] Dienstmann et al., Cancer Discov (2015), v19
[2] Griffith et al., Nat Genet (2017), version 01 June 2017

Patient		Gene-Drug Associations						
Gene	Variant	Disease	Known Variant	Association	Drugs	Evidence	PMID	Level
		melanoma	ampl. (GoF)	no response	imatinib	early trials	23775962	B2
MDM4	ampl.	angiosarcoma	ampl. (GoF)	sensitivity	MDM2/MDMX inhibitors	preclinical	24336067	B3
RB1	del.	retinoblastoma	any (LoF)	sensitivity	MDM2/MDMX inhibitors	preclinical	17080083, 21515735	B3
BRCA2	del.		(LoF)	sensitivity	Mitomycin C	preclinical	16243825	B3
TP53	I162N	bladder	mut. (LoF)	sensitivity	mitomycin C, gemcitabine, doxorubicin	preclinical	27397505	B3
BRCA2	del.	ovarian	(LoF), mut.	sensitivity	Olaparib	clin. trials	23346317,21862407	B2
		prostate	(LoF)	sensitivity	Olaparib	clin. trials	26510020	B2
RB1	del.	breast	(LoF)	resistance or non-response	Palbociclib (PD0332991)	preclinical	20473330	A3
		glioblastoma	(LoF)	resistance or non-response	Palbociclib (PD0332991)	preclinical	20354191	B3
BRCA2	del.	breast	(LoF)	sensitivity	Talazoparib	clin. trials	28242752	A2
		ovarian	(LoF)	sensitivity	Talazoparib	clin. trials	28242752	B2
TP53	I162N	head and neck	any (LoF)	sensitivity	WEE1 inhibitors	preclinical	25125259	B3
		breast	mut. (LoF)	resistance	CDK4/CDK6 inhibitor abemaciclib	early trials	27217383	A2
MCL1	ampl.	unspecified	ampl. (GoF)	resistance	anti-tubulin agents	preclinical	21368834	B3
TP53	I162N	CLL	mut.	sensitivity	Alemtuzumab	clin. trials	14726385	B2
MYC	ampl.	neuroblastoma	ampl. (GoF)	sensitivity	CDK7 inhibitors	preclinical	25416950	B3
TP53	I162N	gastric	mut.	sensitivity	Chemotherapy	clin. trials	24740294	B2
MYC	ampl.	neuroblastoma	ampl. (GoF)	sensitivity	FACT inhibitor	preclinical	26537256	B3
RB1	del.	retinoblastoma	any (LoF)	sensitivity	HDAC inhibitors	preclinical	18483379	B3
TP53	I162N	unspecified	R248Q, R175H (GoF)	sensitivity	HSP90 inhibitors	preclinical	26009011	B3
TOP1	ampl.	colorectal	ampl., expression	sensitivity	Irinotecan	clin. trials	24256029,19775480	B2
BRCA2	del.	ovarian	del. (LoF)	sensitivity	PARP inhibitors	preclinical	22392482	B3
PDGFRA	ampl.	glioblastoma	ampl. (GoF)	no sensitivity	PDGFR inhibitors	preclinical	23544171	B3
MYC	ampl.	prostate	ampl. (GoF)	sensitivity	PIM inhibitors	preclinical	25505253	B3
TP53	I162N	thymic lymphoma	any (LoF)	sensitivity	pramlintide	preclinical	25409149	B3
KIT	ampl.	melanoma	ampl. (GoF)	no response	sunitinib	early trials	22261812	B2
MYC	ampl.	colorectal	ampl. (GoF)	sensitivity	temozolomide	preclinical	27397505	B3

Other genes: here you can find other genes that might be interesting to check (information from Target DB[3] and Meric-Bernstam list[4]). No level information is provided in this section.

Patient		Drug-Gene Interactions		
Gene	Variant	Known Variant	Description	Drugs
FGF1	I331N	Mutation; Amplification	Upstream ligand of FGFR, possible sensitivity to FGFR inhibitors	FGFR Inhibitors
ABL2	amp	Mutation; Amplification		Treatment with ABL2 inhibitors
SRC	amp	Mutation; Amplification		Treatment with SRC inhibitors

[3] Van Allen et al., Nat Med (2014), v3
[4] Meric-Bernstam et al., J Natl Cancer Inst (2015)

MTB Report - From somatic variants to treatment options

Department of Medical Statistics, University Medical Center Goettingen

September 27, 2017

PATIENT INFORMATION

**Patient ID**	MASTER-02	**Tissue Type**	?
**Gender**	Male	**Tumor Content (%)**	90
**Disease**	Pancreas carcinoma	**Number SNVs**	49
**Previous Therapies**	-	**Number CNVs**	1433
**Tumor Board Decision**	-	**Number Fusions**	1

GENE-DRUG PREDICTIVE ASSOCIATIONS

Method: Somatic variants of the patient (mutations, amplifications, deletions, rearrangments) are searched in curated databases of predictive biomarkers (GKDB[1], CIViC[2]) and reported according to their clinical evidence (see Levels of Evidence). In the following two tables (SNVs and CNVs), basic information of the somatic variants with relevant clinical implications can be found:

Gene	Patient's Variant	Level of Evidence	Variant Freq.	Zygosity	Quality (Phred)
KRAS	G12D	**B1** ,**A2** ,**B2** ,**A3** ,**B3**	0.40	het	225

Gene	Patient's Variant	Level of Evidence	Seg. Mean	Size (Mb)
CCND2	ampl.	**B3**	0.53	9.3
CDKN2A	del.	**B2** ,**B3**	-1.45	0.6
CDKN2B	del.	**B3**	-1.45	0.6
FGFR1	ampl.	**B2** ,**B3**	0.92	4.9
KRAS	G12D,ampl.	**B1** ,**A2** ,**B2** ,**A3** ,**B3**	0.52	21.1
MTAP	del.	**B3**	-1.45	0.6
MYC	ampl.	**B3**	1.02	13.6
SMAD4	del.	**B2**	-1.37	0.4
TYMS	ampl.	**B2**	0.51	3.0

Levels of Evidence: Findings are classified into 6 levels of evidence combining the **axis A-B** and the **axis 1-2-3**. Level A means evidence in the same cancer type. Level B means evidence in any other cancer type. On the 1-2-3 axis, level 1 means evidence supported by drug approval organizations or clinical guidelines, level 2 contains clinical evidence (clinical trials, case reports) and level 3 consists of preclinical evidence. The distribution of findings into levels is summarized in the right figure

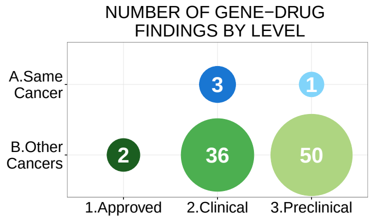

Table of Results: All the predicitve associations are detailed in this table. The results are sorted by 1) drug frequency, 2) levels of evidence (A1-B1-A2-B2-A3-B3). To allow a quick interpretation, the type of association (response, resistance) is colored (green, red) and new variants are gray and underlined.

Patient Gene	Variant	Disease	Known Variant	Gene-Drug Association	Associations Drugs	Evidence	PMID	Level
KRAS	G12D	pancreatic	any (GoF)	response	gemcitabine + MEK inhibitors	early trials	23583440	A2
		pancreatic	any (GoF)	no response	PI3K pathway inhibitors + MEK inhibitor	early trials	ASCO 2015 (abstr 4119)	A2
		lung	any (GoF)	response	MEK inhibitors	early trials	23200175, 24947927, 25667274, 25722381	B2
		lung	any (GoF)	response	PI3K pathway inhibitors + MEK inhibitors	early trials	25516890	B2
		biliary tract	any (GoF)	response	MEK inhibitors	early trials	23391555	B2
		colorectal	ampl. (GoF)	resistance	BRAF inhibitor + MEK inhibitor/anti-EGFR mAb	case report	ENA 2014 (abstr 428)	B2
		colorectal	any (GoF)	sensitivity	MEK inhibitors + PI3K pathway inhibitors	preclinical	22392911	B3
		colorectal	any (GoF)	sensitivity	MEK inhibitors + BCL-XL inhibitors	preclinical	23245996	B3
		colorectal	any (GoF)	sensitivity	MEK inhibitors + IGF1R inhibitors	preclinical	24045180	B3
		AML	any (GoF)	sensitivity	MEK inhibitors	preclinical	22507781	B3
		cervical	any (GoF)	sensitivity	MEK inhibitors	preclinical	22169769	B3
		endometrial	any (GoF)	sensitivity	PI3K pathway inhibitors + MEK inhibitors	preclinical	21984976, 22662154	B3

[1]Dienstmann et al., Cancer Discov (2015), v19
[2]Griffith et al., Nat Genet (2017), version 01 June 2017

Patient		Gene-Drug Associations						
Gene	Variant	Disease	Known Variant	Association	Drugs	Evidence	PMID	Level
		myeloma	any (GoF)	sensitivity	PI3K pathway inhibitors + MEK inhibitors	preclinical	22985491	B3
CDKN2A	del.	breast	loss	sensitivity	Letrozole,Palbociclib (PD0332991)	case report	26715889	B2
		bone ewing sarcoma	loss	sensitivity	Linsitinib,Palbociclib	preclinical	27286459	B3
		dermatofibro-sarcoma	loss	sensitivity	Palbociclib	preclinical	25852058	B3
		ovarian	loss	sensitivity	Palbociclib (PD0332991)	preclinical	21278246	B3
		renal	loss	sensitivity	Palbociclib (PD0332991)	preclinical	23898052	B3
		melanoma	loss	sensitivity	Palbociclib (PD0332991)	preclinical	24495407	B3
CDKN2B	del.	renal	loss	sensitivity	Palbociclib (PD0332991)	preclinical	23898052	B3
		bone ewing sarcoma	loss	sensitivity	Palbociclib,Linsitinib	preclinical	27286459	B3
CDKN2A	del.	melanoma	any (LoF)	response	CDK4/6 inhibitors	case report	ASCO 2013 (abstr 2500)	B2
CCND2	ampl.	unspecified	ampl. (GoF)	sensitivity	CDK4/6 inhibitors	preclinical	22471707	B3
CDKN2A	del.	unspecified	any (LoF)	sensitivity	CDK4/6 inhibitors	preclinical	22471707	B3
		glioblastoma	any (LoF)	sensitivity	CDK4/6 inhibitors	preclinical	22586120, 22711607	B3
CDKN2B	del.	unspecified	any (LoF)	sensitivity	CDK4/6 inhibitors	preclinical	22471707	B3
		glioblastoma	any (LoF)	sensitivity	CDK4/6 inhibitors	preclinical	22711607	B3
KRAS	G12D	endometrial	any (GoF)	resistance	PI3K pathway inhibitors	preclinical	22662154	B3
		lung	mut.	resistance or non-response	Docetaxel,Selumetinib	clin. trials	28492898	B2
		lung	any (GoF)	no response	selumetinib	early trials	26802155	B2
		colorectal	mut.	sensitivity	RAF265,Selumetinib (AZD6244)	preclinical	25199829	B3
		lung	mut.	sensitivity	RAF265,Selumetinib (AZD6244)	preclinical	25199829	B3
		colorectal	mut.	sensitivity	Selumetinib (AZD6244),R1507	preclinical	21985784	B3
		lung	mut.	sensitivity	Trametinib	clin. trials	22805291	B2
		lung	mut.	sensitivity	Trametinib,Docetaxel	clin. trials	25722381	B2
		colorectal	mut.	sensitivity	Afatinib,Trametinib	preclinical	24685132	B3
		lung	mut.	sensitivity	Afatinib,Trametinib	preclinical	24685132	B3
		breast	mut.	sensitivity	Trametinib	preclinical	22169769	B3
		lung	any (GoF)	sensitivity	BET inhibitors in LKB1 wt	preclinical	23129625, 24045185	B3
MYC	ampl.	myeloma	ampl. (GoF)	sensitivity	BET inhibitors	preclinical	21889194	B3
		neuroblastoma	ampl. (GoF)	sensitivity	BET inhibitors	preclinical	23430699	B3
KRAS	ampl.	melanoma	ampl.	sensitivity	Carboplatin,Docetaxel,Sorafenib	clin. trials	26307133	B2
		lung	mut.	sensitivity	Farnesylthiosalicylic Acid	clin. trials	21847063	B2
		lung	any (GoF)	response	fatty acid synthase inhibitors	case report	AACR 2016, abstr LB214	B2
FGFR1	ampl.	breast	ampl.	sensitivity	BGJ-398	clin. trials	27870574	B2
		bladder	ampl.	sensitivity	BGJ-398	case report	27870574	B2
KRAS	G12D	colorectal	any (GoF)	resistance	cetuximab, panitumumab	FDA-rejected	FDA	B1
		colorectal	mut.	sensitivity	Cetuximab,Dasatinib	preclinical	20956938	B3
		lung	any (GoF)	resistance	EGFR TKIs	NCCN guidelines	20921461	B1
		gastric	any (GoF)	resistance	anti-EGFR mAbs	preclinical	22614881, 22290393	B3
		colorectal	mut.	sensitivity	IMO,EGFR Inhibitor	preclinical	21890455	B3
FGFR1	ampl.	lung (squa.)	ampl. (GoF)	response	FGFR inhibitors	early trials	AACR 2012 (abstract LB-122), AACR 2013 (abstract LB-145)	B2
		breast	ampl. (GoF)	response	FGFR inhibitors	early trials	25193991	B2
KRAS	G12D		G12A, G12D, G12C, G12S	resistance or non-response	Melphalan	clin. trials	19284554	B2
			G12A, G12D, G12C, G12S	resistance or non-response	Melphalan	preclinical	11050000,12483530,16497971	B3
		lung	any (GoF)	response	pan-RAF inhibitors	case report	AACR 2016 (abstr CT005)	B2
		endometrial	any (GoF)	response	pan-RAF inhibitors	case report	AACR 2016 (abstr CT005)	B2

Patient		Gene-Drug Associations						
Gene	Variant	Disease	Known Variant	Association	Drugs	Evidence	PMID	Level
		colorectal	G12A, G13D, G12V, G12D, G12C, G12R, G12S	resistance or non-response	Panitumumab	case report	18316791	B2
FGFR1	ampl.	lung	ampl.	sensitivity	PD173074	preclinical	21160078	B3
		lung	ampl.	sensitivity	PD173074	preclinical	21666749	B3
		bone ewing sarcoma	ampl.	sensitivity	Ponatinib	preclinical	26179511	B3
		breast	ampl.	sensitivity	Ponatinib	preclinical	22238366	B3
KRAS	G12D	colorectal	mut.	n/a	RO4987655	clin. trials	24947927	B2
		lung	mut.	n/a	RO4987655	clin. trials	24947927	B2
		hepatocellular	mut.	sensitivity	Sorafenib,Refametinib	clin. trials	25294897	B2
FGFR1	ampl.	breast	ampl.	resistance or non-response	4-hydroxytamoxifen	preclinical	20179196	B3
MTAP	del.	breast	del.	sensitivity	5-Fluoropyrimidine	preclinical	26751376	B3
SMAD4	del.	colorectal	del.	resistance or non-response	5-fluorouracil	clin. trials	12237773	B2
KRAS	G12D	colorectal	mut.	sensitivity	BAY 86-9766	case report	23434733	B2
		lung	any (GoF)	response	CDK4/CDK6 inhibitor abemaciclib	early trials	27217383	B2
		colorectal	G12D	sensitivity	Adoptive T-cell Transfer	case report	27959684	B2
		lung	G12D	sensitivity	ARRY-142886,BEZ235 (NVP-BEZ235, Dactolisib)	preclinical	19029981	B3
		lung	mut.	sensitivity	Atezolizumab,Nivolumab	clin. trials	28525386	B2
		breast	mut.	sensitivity	AZD5438	preclinical	26881434	B3
		pancreatic	mut.	sensitivity	SCH772984,AZD8186	preclinical	26725216	A3
		colorectal	mut.	resistance or non-response	Chemotherapy,Bevacizumab	clin. trials	23828442	B2
FGFR1	ampl.	lung	ampl.	sensitivity	BGJ398	clin. trials	27870574	B2
KRAS	G12D	colorectal	any (GoF)	sensitivity	mTOR inhibitors + BH3 mimetics	preclinical	24163374	B3
MYC	ampl.	neuroblastoma	ampl. (GoF)	sensitivity	CDK7 inhibitors	preclinical	25416950	B3
KRAS	G12D	ovarian	mut.	sensitivity	Decitabine	preclinical	25968887	B3
FGFR1	ampl.	breast	ampl.	sensitivity	Dovitinib	clin. trials	23658459	B2
MYC	ampl.	neuroblastoma	ampl. (GoF)	sensitivity	FACT inhibitor	preclinical	26537256	B3
CDKN2A	del.	melanoma	loss	sensitivity	Flavopiridol	preclinical	12777976	B3
KRAS	G12D	breast	mut.	sensitivity	GDC-0623,G-573	preclinical	23934108	B3
		lung	G12A, G12D, G12C, G12S	resistance or non-response	Gefitinib	clin. trials	17409929	B2
		colorectal	mut.	resistance or non-response	Ixazomib	preclinical	26709701	B3
		pancreatic	G12D	sensitivity	MK-2206	case report	22025163	A2
TYMS	ampl.	lung	ampl.	resistance or non-response	Pemetrexed	clin. trials	23645741	B2
MYC	ampl.	prostate	ampl. (GoF)	sensitivity	PIM inhibitors	preclinical	25505253	B3
KRAS	G12D	endometrial	mut.	sensitivity	Ridaforolimus,Temsirolimus	clin. trials	24166148	B2
MYC	ampl.	colorectal	ampl. (GoF)	sensitivity	temozolomide	preclinical	27397505	B3
KRAS	G12D		G12D	resistance or non-response	Vemurafenib	case report	26352686	B2

Other genes: here you can find other genes that might be interesting to check (information from Target DB[3] and Meric-Bernstam list[4]). No level information is provided in this section.

[1] ”No other genes found”

[3] Van Allen et al., Nat Med (2014), v3
[4] Meric-Bernstam et al., J Natl Cancer Inst (2015)

MTB Report - From somatic variants to treatment options

Department of Medical Statistics, University Medical Center Goettingen

September 27, 2017

PATIENT INFORMATION

**Patient ID**	MASTER-03	**Tissue Type**	?
**Gender**	Female	**Tumor Content (%)**	NA
**Disease**	Leiomyosarcoma of retroperitoneum with lung metastasis	**Number SNVs**	31
**Previous Therapies**	Doxorubicin, Ifosfamid	**Number CNVs**	3964
**Tumor Board Decision**	PTPRJ deletion : Pazopanib; CDK12 + BRCA2 deletions : Cisplatin	**Number Fusions**	6

GENE-DRUG PREDICTIVE ASSOCIATIONS

Method: Somatic variants of the patient (mutations, amplifications, deletions, rearrangments) are searched in curated databases of predictive biomarkers (GKDB[1], CIViC[2]) and reported according to their clinical evidence (see Levels of Evidence). In the following two tables (SNVs and CNVs), basic information of the somatic variants with relevant clinical implications can be found:

Gene	Patient's Variant	Level of Evidence	Variant Freq.	Zygosity	Quality (Phred)

Gene	Patient's Variant	Level of Evidence	Seg. Mean	Size (Mb)
BRCA2	del.	B2 ,B3	-0.81	3.7
FANCA	del.	B2	-0.66	43.8
HSPH1	del.	B2	-0.81	3.7
RB1	del.	B3	-0.64	45.0

Levels of Evidence: Findings are classified into 6 levels of evidence combining the **axis A-B** and the **axis 1-2-3**. Level A means evidence in the same cancer type. Level B means evidence in any other cancer type. On the 1-2-3 axis, level 1 means evidence supported by drug approval organizations or clinical guidelines, level 2 contains clinical evidence (clinical trials, case reports) and level 3 consists of preclinical evidence. The distribution of findings into levels is summarized in the right figure

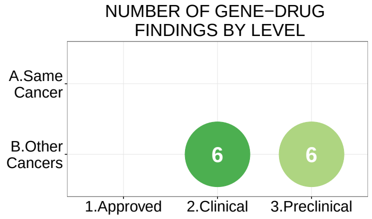

Table of Results: All the predicitve associations are detailed in this table. The results are sorted by 1) drug frequency, 2) levels of evidence (A1-B1-A2-B2-A3-B3). To allow a quick interpretation, the type of association (response, resistance) is colored (green, red) and new variants are gray and underlined.

Patient		Gene-Drug Associations						
**Gene**	**Variant**	**Disease**	**Known Variant**	**Association**	**Drugs**	**Evidence**	**PMID**	**Level**
BRCA2	del.	ovarian	(LoF), mut.	sensitivity	Olaparib	clin. trials	23346317,21862407	B2
		prostate	(LoF)	sensitivity	Olaparib	clin. trials	26510020	B2
RB1	del.	breast	(LoF)	resistance or non-response	Palbociclib (PD0332991)	preclinical	20473330	B3
		glioblastoma	(LoF)	resistance or non-response	Palbociclib (PD0332991)	preclinical	20354191	B3
FANCA	del.	prostate	any (LoF)	response	PARP inhibitors	early trials	26510020	B2
BRCA2	del.	ovarian	del. (LoF)	sensitivity	PARP inhibitors	preclinical	22392482	B3
		breast	(LoF)	sensitivity	Talazoparib	clin. trials	28242752	B2
		ovarian	(LoF)	sensitivity	Talazoparib	clin. trials	28242752	B2
HSPH1	del.	colorectal	T17 del.	sensitivity	Oxaliplatin,5-fluorouracil	clin. trials	24512910	B2
RB1	del.	retinoblastoma	any (LoF)	sensitivity	HDAC inhibitors	preclinical	18483379	B3
		retinoblastoma	any (LoF)	sensitivity	MDM2/MDMX inhibitors	preclinical	17080083, 21515735	B3
BRCA2	del.		(LoF)	sensitivity	Mitomycin C	preclinical	16243825	B3

Other genes: here you can find other genes that might be interesting to check (information from Target DB[3] and Meric-Bernstam list[4]). No level information is provided in this section.

Patient		Drug-Gene Interactions		
**Gene**	**Variant**	**Known Variant**	**Description**	**Drugs**

[1] Dienstmann et al., Cancer Discov (2015), v19
[2] Griffith et al., Nat Genet (2017), version 01 June 2017
[3] Van Allen et al., Nat Med (2014), v3
[4] Meric-Bernstam et al., J Natl Cancer Inst (2015)

Patient		Drug-Gene Interactions		
**Gene**	**Variant**	**Known Variant**	**Description**	**Drugs**
ESR1	amp	Amplification; Mutations	Amplification may predict ER positivity in breast cancer. Mutation may predict resistance to endocrine therapy in breast cancer.	Hormonal therapy
MAP3K4	amp	Mutation; Amplification		Treatment with JNK1 inhibitor

MTB Report - From somatic variants to treatment options

Department of Medical Statistics, University Medical Center Goettingen

September 27, 2017

PATIENT INFORMATION

**Patient ID**	MASTER-04	**Tissue Type**	?
**Gender**	Female	**Tumor Content (%)**	20
**Disease**	Ovarian carcinoma metastasis	**Number SNVs**	98
**Previous Therapies**	-	**Number CNVs**	3555
**Tumor Board Decision**	TSC2 stopgain : mTOR-Inhibitor (Everolimus)	**Number Fusions**	16

GENE-DRUG PREDICTIVE ASSOCIATIONS

Method: Somatic variants of the patient (mutations, amplifications, deletions, rearrangments) are searched in curated databases of predictive biomarkers (GKDB[1], CIViC[2]) and reported according to their clinical evidence (see Levels of Evidence). In the following two tables (SNVs and CNVs), basic information of the somatic variants with relevant clinical implications can be found:

Gene	Patient's Variant	Level of Evidence	Variant Freq.	Zygosity	Quality (Phred)
BCOR	S1024T	B3	0.18	het	88
TP53	Y236C	A2 ,B2 ,B3	0.79	het	225
TSC2	R505X	B2 ,B3	0.42	het	221

Gene	Patient's Variant	Level of Evidence	Seg. Mean	Size (Mb)
AURKA	ampl.	B3	0.58	39.2
BIRC7	ampl.	B3	0.58	39.2
BRCA1	del.	A2 ,B2 ,A3 ,B3	-0.84	2.6
CCNE1	ampl.	A3 ,B3	0.73	15.2
CDK12	del.	A3	-0.87	1.9
FANCA	del.	B2	-0.71	59.8
FRS2	ampl.	B3	0.73	6.5
MDM2	ampl.	B2	0.73	6.5
NF1	del.	B2 ,B3	-0.85	2.6
PALB2	del.	B2 ,B3	-0.70	17.5
RICTOR	ampl.	B2	1.56	12.5
SUZ12	del.	B3	-0.87	4.5
TOP1	ampl.	B2	0.58	39.2
TSC2	R505X,del.	B2 ,B3	-0.78	11.2

Levels of Evidence: Findings are classified into 6 levels of evidence combining the **axis A-B** and the **axis 1-2-3**. Level A means evidence in the same cancer type. Level B means evidence in any other cancer type. On the 1-2-3 axis, level 1 means evidence supported by drug approval organizations or clinical guidelines, level 2 contains clinical evidence (clinical trials, case reports) and level 3 consists of preclinical evidence. The distribution of findings into levels is summarized in the right figure

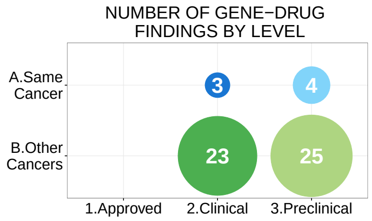

Table of Results: All the predicitve associations are detailed in this table. The results are sorted by 1) drug frequency, 2) levels of evidence (A1-B1-A2-B2-A3-B3). To allow a quick interpretation, the type of association (response, resistance) is colored (green, red) and new variants are gray and underlined.

Patient		Gene-Drug			Associations			
**Gene**	**Variant**	**Disease**	**Known Variant**	**Association**	**Drugs**	**Evidence**	**PMID**	**Level**
FANCA	del.	prostate	any (LoF)	response	PARP inhibitors	early trials	26510020	B2
PALB2	del.	prostate	any (LoF)	response	PARP inhibitors	early trials	AACR 2015 (abstr CT322), 26510020	B2
BRCA1	del.	ovarian	del. (LoF)	sensitivity	PARP inhibitors	preclinical	22392482	A3
CDK12	del.	ovarian	any (LoF)	sensitivity	PARP inhibitors	preclinical	24240700, 24554720	A3
PALB2	del.	pancreatic	any (LoF)	sensitivity	PARP inhibitors	preclinical	25263539, NCT01585805	B3
TSC2	R505X	angiomyolipoma	any (LoF)	response	mTOR inhibitors	early trials	23312829, 21525172, 20048174	B2
RICTOR	ampl.	lung	ampl. (GoF)	response	mTORC1/2 inhibitors	case report	26370156	B2
NF1	del.	neurosarcoma	any (LoF)	sensitivity	mTOR inhibitors	preclinical	18483311, 20505189, 24509877	B3

[1]Dienstmann et al., Cancer Discov (2015), v19
[2]Griffith et al., Nat Genet (2017), version 01 June 2017

Patient		Gene-Drug Associations						
Gene	Variant	Disease	Known Variant	Association	Drugs	Evidence	PMID	Level
		neurosarcoma	any (LoF)	sensitivity	mTOR inhibitors + HSP90 inhibitors	preclinical	21907929	B3
		glioblastoma	any (LoF)	sensitivity	mTOR inhibitors	preclinical	19573811	B3
		melanoma	any (LoF)	resistance	selumetinib in BRAF mutant tumors	case report	23444215, 23288408	B2
		melanoma	any (LoF)	resistance	BRAF inhibitors in BRAF mutant tumor	preclinical	23288408, 24576830	B3
MDM2	ampl.		ampl. (GoF)	resistance	cisplatin	early trials	27646943	B2
TP53	Y236C		mut. (LoF)	resistance	cisplatin	early trials	27646943	B2
BIRC7	ampl.	colorectal	ampl.	resistance or non-response	Cisplatin	preclinical	23188704	B3
NF1	del.	neurosarcoma	any (LoF)	no response	everolimus + bevacizumab	early trials	ASCO 2016 (abstr 11053)	B2
TSC2	R505X	thyroid	any (LoF)	response	everolimus	case report	25295501	B2
		gastric	any (LoF)	response	everolimus	case report	26859683	B2
BRCA1	del.	ovarian	(LoF), mut.	sensitivity	Olaparib	clin. trials	23346317,21862407	A2
		prostate	(LoF)	sensitivity	Olaparib	case report	26510020	B2
CDK12	del.	ovarian	(LoF)	sensitivity	Olaparib	preclinical	24240700	A3
AURKA	ampl.	unspecified	ampl. (GoF)	sensitivity	AURK inhibitors	preclinical	22302096	B3
		prostate	ampl. (GoF)	sensitivity	AURK inhibitors	preclinical	22389870	B3
TP53	Y236C	AML	mut. (LoF)	response	decitabine	early trials	27959731	B2
		MDS	mut. (LoF)	response	decitabine	early trials	27959731	B2
		lung	mut.	resistance or non-response	Docetaxel	preclinical	22425996	B3
		lung	mut.	sensitivity	Docetaxel,Selumetinib (AZD6244)	preclinical	22425996	B3
		unspecified	R248Q, R175H (GoF)	sensitivity	HSP90 inhibitors	preclinical	26009011	B3
TSC2	R505X	lymphangio-leiomyomatosis	any (LoF)	sensitivity	SRC inhibitors	preclinical	24691995	B3
		lymphangio-leiomyomatosis	any (LoF)	sensitivity	SRC inhibitors	preclinical	24691995	B3
BRCA1	del.	ovarian	(LoF)	sensitivity	Talazoparib	clin. trials	28242752	A2
		breast	(LoF)	sensitivity	Talazoparib	clin. trials	28242752	B2
TP53	Y236C	ovarian	any (GoF)	response	WEE1 inhibitors + carboplatin	early trials	ASCO2015 (abstr 2507)	A2
		head and neck	any (LoF)	sensitivity	WEE1 inhibitors	preclinical	25125259	B3
		breast	mut. (LoF)	resistance	CDK4/CDK6 inhibitor abemaciclib	early trials	27217383	B2
		CLL	mut.	sensitivity	Alemtuzumab	clin. trials	14726385	B2
BCOR	S1024T	gastric	mut. (LoF)	sensitivity	enzastaurin (PKCbeta inhibitor)	preclinical	27397505	B3
SUZ12	del.	unspecified	any (LoF)	sensitivity	BET inhibitors	preclinical	25119042	B3
RICTOR	ampl.	lung	ampl.	sensitivity	MLN0128,Cc-223	case report	26370156	B2
CCNE1	ampl.	unspecified	ampl. (GoF)	sensitivity	CDK2 inhibitors	preclinical	22471707	B3
TP53	Y236C	gastric	mut.	sensitivity	Chemotherapy	clin. trials	24740294	B2
NF1	del.	malignant peripheral nerve sheath tumor	loss	sensitivity	JQ1 Compound	preclinical	24373973	B3
BRCA1	del.	breast	(LoF)	sensitivity	CX-5461,CX-3543	preclinical	28211448	B3
CCNE1	ampl.	ovarian	ampl.	sensitivity	Dinaciclib,MK-2206	preclinical	27663592	A3
TP53	Y236C	bladder	mut. (LoF)	sensitivity	mitomycin C, gemcitabine, doxorubicin	preclinical	27397505	B3
FRS2	ampl.	liposarcoma	ampl. (GoF)	sensitivity	FGFR inhibitors	preclinical	23393200	B3
TOP1	ampl.	colorectal	ampl., expression	sensitivity	Irinotecan	clin. trials	24256029,19775480	B2
MDM2	ampl.	liposarcoma	ampl. (GoF)	response	MDM2 inhibitors (tumors TP53 wt)	early trials	23084521, ASCO 2015 (abstr 10564)	B2
NF1	del.	neurosarcoma	any (LoF)	sensitivity	MEK inhibitors	preclinical	23221341	B3
PALB2	del.	pancreatic	any (LoF)	response	Mytomycin C	case report	21135251	B2
CCNE1	ampl.	breast	ampl.	resistance or non-response	Palbociclib (PD0332991)	preclinical	27020857	B3
NF1	del.	melanoma	any (LoF)	response	PD1 blockade	early trials	ASCO 2016 (abstr 105)	B2
PALB2	del.	pancreatic	any (LoF)	response	platinum	case report	25719666	B2
TP53	Y236C	thymic lymphoma	any (LoF)	sensitivity	pramlintide	preclinical	25409149	B3
NF1	del.	melanoma	any (LoF)	sensitivity	trametinib	preclinical	24576830	B3

Other genes: here you can find other genes that might be interesting to check (information from Target DB[3] and Meric-Bernstam list[4]). No level information is provided in this section.

Patient		Drug-Gene Interactions		
**Gene**	**Variant**	**Known Variant**	**Description**	**Drugs**
SRC	amp	Mutation; Amplification		Treatment with SRC inhibitors

[3]Van Allen et al., Nat Med (2014), v3
[4]Meric-Bernstam et al., J Natl Cancer Inst (2015)

MTB Report - From somatic variants to treatment options

Department of Medical Statistics, University Medical Center Goettingen

September 27, 2017

PATIENT INFORMATION

**Patient ID**	MASTER-05	**Tissue Type**	?
**Gender**	Female	**Tumor Content (%)**	100
**Disease**	Myoxid liposarcoma	**Number SNVs**	11
**Previous Therapies**	Doxorubicin, Dacarbacin	**Number CNVs**	107
**Tumor Board Decision**	PIK3CA and PTEN missense mutations: mTOR inhibitor, AKT inhibitor, PI3K inhibitor	**Number Fusions**	2

GENE-DRUG PREDICTIVE ASSOCIATIONS

Method: Somatic variants of the patient (mutations, amplifications, deletions, rearrangments) are searched in curated databases of predictive biomarkers (GKDB[1], CIViC[2]) and reported according to their clinical evidence (see Levels of Evidence). In the following two tables (SNVs and CNVs), basic information of the somatic variants with relevant clinical implications can be found:

Gene	Patient's Variant	Level of Evidence	Variant Freq.	Zygosity	Quality (Phred)
PIK3CA	C420R	B2 ,B3	0.39	het	219
PTEN	R130G	B2 ,B3	0.71	het	225

Gene	Patient's Variant	Level of Evidence	Seg. Mean	Size (Mb)
CDKN2A	del.	B2 ,B3	-1.35	1.0
CDKN2B	del.	B3	-1.35	1.0
MTAP	del.	B3	-1.35	1.0

Levels of Evidence: Findings are classified into 6 levels of evidence combining the **axis A-B** and the **axis 1-2-3**. Level A means evidence in the same cancer type. Level B means evidence in any other cancer type. On the 1-2-3 axis, level 1 means evidence supported by drug approval organizations or clinical guidelines, level 2 contains clinical evidence (clinical trials, case reports) and level 3 consists of preclinical evidence. The distribution of findings into levels is summarized in the right figure

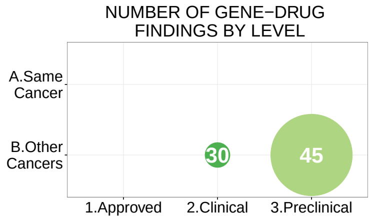

Table of Results: All the predicitve associations are detailed in this table. The results are sorted by 1) drug frequency, 2) levels of evidence (A1-B1-A2-B2-A3-B3). To allow a quick interpretation, the type of association (response, resistance) is colored (green, red) and new variants are gray and underlined.

Patient		Gene-Drug Associations						
**Gene**	**Variant**	**Disease**	**Known Variant**	**Association**	**Drugs**	**Evidence**	**PMID**	**Level**
PIK3CA	C420R	breast	any (GoF)	response	PI3K aplha inhibitors	early trials	AACR 2013 (abstr LB-64), ESMO 2013 (abstr P017)	B2
		breast	any (GoF)	response	PI3K pathway inhibitors	early trials	22271473	B2
		cervical	any (GoF)	response	PI3K pathway inhibitors	early trials	22271473, ASCO 2013 (abstr 2531)	B2
		endometrial	any (GoF)	response	PI3K pathway inhibitors	early trials	22271473, 27672108	B2
		ovarian	any (GoF)	response	PI3K pathway inhibitors	early trials	22271473, AACR 2013 (abstr LB-66), 25231405	B2
		gastric	any (GoF)	response	PI3K alpha inhibitors	case report	ASCO 2015 (abstr 2501)	B2
		bladder	any (GoF)	response	PI3K pathway inhibitors	case report	ASCO 2015 (abstr 2516)	B2
		head and neck	any (GoF)	response	PI3K pathway inhibitors	case report	26787751, 26763254	B2
		lung	any (GoF)	response	PI3K pathway inhibitors	case report	ESMO 2013 (abstr P017)	B2
PTEN	R130G	prostate	any (LoF)	response	PI3K beta inhibitor	case report	ASCO 2014 (abstr 2514)	B2
PIK3CA	C420R	colorectal	any (GoF)	sensitivity	PI3K pathway inhibitors (alone or in combination)	preclinical	23475782, 22392911	B3

[1]Dienstmann et al., Cancer Discov (2015), v19
[2]Griffith et al., Nat Genet (2017), version 01 June 2017

Patient		Gene-Drug	Associations					
Gene	Variant	Disease	Known Variant	Association	Drugs	Evidence	PMID	Level
		lung	any (GoF)	sensitivity	PI3K pathway inhibitors (alone or in combination)	preclinical	23136191	B3
		glioblastoma	any (GoF)	sensitivity	PI3K pathway inhibitors	preclinical	19671762	B3
		thyroid	any (GoF)	sensitivity	PI3K pathway inhibitors	preclinical	21289267	B3
		breast	mut.	sensitivity	PI3K Inhibitor,Palbociclib (PD0332991)	preclinical	25002028	B3
		breast	mut.	sensitivity	PI3K Inhibitor,Ribociclib	preclinical	25002028	B3
PTEN	R130G	prostate	any (LoF)	sensitivity	PI3K pathway inhibitors + AR antagonists	preclinical	21575859	B3
		endometrial	any (LoF)	sensitivity	PI3K pathway inhibitors	preclinical	22662154	B3
		breast	any (LoF)	sensitivity	PI3K pathway inhibitors	preclinical	23085766, 22932669	B3
		thyroid	any (LoF)	sensitivity	PI3K pathway inhibitors	preclinical	21289267	B3
		ovarian	any (LoF)	sensitivity	PI3K pathway inhibitors	preclinical	21632463	B3
		ovarian	any (LoF)	sensitivity	PI3K pathway inhibitors + MEK inhibitors	preclinical	21632463	B3
		glioblastoma	any (LoF)	sensitivity	PI3K pathway inhibitors (alone or in combination)	preclinical	21325073, 21191045, 17804702	B3
		lung	any (LoF)	sensitivity	PI3K pathway inhibitors (alone or in combination)	preclinical	23136191	B3
CDKN2A	del.	breast	loss	sensitivity	Letrozole,Palbociclib (PD0332991)	case report	26715889	B2
		bone ewing sarcoma	loss	sensitivity	Linsitinib,Palbociclib	preclinical	27286459	B3
		dermatofibro-sarcoma	loss	sensitivity	Palbociclib	preclinical	25852058	B3
		ovarian	loss	sensitivity	Palbociclib (PD0332991)	preclinical	21278246	B3
		renal	loss	sensitivity	Palbociclib (PD0332991)	preclinical	23898052	B3
		melanoma	loss	sensitivity	Palbociclib (PD0332991)	preclinical	24495407	B3
CDKN2B	del.	renal	loss	sensitivity	Palbociclib (PD0332991)	preclinical	23898052	B3
		bone ewing sarcoma	loss	sensitivity	Palbociclib,Linsitinib	preclinical	27286459	B3
PIK3CA	C420R	breast	any (GoF)	response	everolimus + trastuzumab + chemotherapy (HER2 ampl)	late trials	27091708	B2
		breast	mut.	sensitivity	Everolimus	clin. trials	27091708	B2
PTEN	R130G	breast	any (LoF)	response	everolimus + trastuzumab + chemotherapy (HER2 ampl)	late trials	27091708	B2
		endometrial	mut.	sensitivity	Everolimus	clin. trials	23238879	B2
		prostate	any (LoF)	response	everolimus	early trials	23582881	B2
PIK3CA	C420R	breast	mut.	sensitivity	Everolimus,PP242	preclinical	21358673	B3
CDKN2A	del.	melanoma	any (LoF)	response	CDK4/6 inhibitors	case report	ASCO 2013 (abstr 2500)	B2
		unspecified	any (LoF)	sensitivity	CDK4/6 inhibitors	preclinical	22471707	B3
		glioblastoma	any (LoF)	sensitivity	CDK4/6 inhibitors	preclinical	22586120, 22711607	B3
CDKN2B	del.	unspecified	any (LoF)	sensitivity	CDK4/6 inhibitors	preclinical	22471707	B3
		glioblastoma	any (LoF)	sensitivity	CDK4/6 inhibitors	preclinical	22711607	B3
PIK3CA	C420R	endometrial	mut.	sensitivity	Pictilisib	preclinical	23674493	B3
		breast	mut.	sensitivity	Pictilisib,17-AAG	preclinical	25855885	B3
		head and neck	mut.	sensitivity	Pictilisib,Trametinib,17-AAG	preclinical	25855885	B3
PTEN	R130G	head and neck	mut.	sensitivity	Pictilisib,17-AAG	preclinical	25855885	B3
PIK3CA	C420R	breast	mut.	sensitivity	AZD5363	preclinical	22294718	B3
		breast	mut.	sensitivity	AZD5363	preclinical	22294718	B3
		gastric	mut.	sensitivity	AZD5363	preclinical	24088382	B3
PTEN	R130G	melanoma	any (LoF)	resistance	BRAF inhibitors in BRAF mutant tumor	preclinical	21317224, 21725359	B3
		melanoma	any (LoF)	resistance	MEK inhibitors in BRAF mutant tumors	preclinical	23039341	B3
PIK3CA	C420R	breast	mut.	resistance or non-response	Trastuzumab	clin. trials	17936563	B2
		breast	any (GoF)	response	AKT inhibitors	early trials	ASCO 2015 (abstr 2500)	B2
PTEN	R130G	pancreatic	any (LoF)	response	AKT inhibitors	case report	22025163	B2
PIK3CA	C420R	breast	mut.	sensitivity	BYL719 (Alpelisib)	preclinical	24608574	B3
PTEN	R130G	prostate	mut.	sensitivity	BYL719 (Alpelisib),AZD8186,Enzalutamide	preclinical	25544636	B3
PIK3CA	C420R	colorectal	mut.	resistance or non-response	Anti-EGFR Monoclonal Antibody	clin. trials	23435830	B2

Patient				Gene-Drug	Associations			
**Gene**	**Variant**	**Disease**	**Known Variant**	**Association**	**Drugs**	**Evidence**	**PMID**	**Level**
PTEN	R130G	colorectal	any (LoF)	resistance	anti-EGFR mAbs	late trials	21163703, 19398573	**B2**
PIK3CA	C420R	colorectal	any (GoF)	resistance	cetuximab	late trials	19223544, 20619739	**B2**
		colorectal	mut.	resistance or non-response	Cetuximab	preclinical	22586653	**B3**
		head and neck	mut.	sensitivity	Taselisib (GDC-0032),Radiation	preclinical	26589432	**B3**
PTEN	R130G	head and neck	mut.	resistance or non-response	Taselisib (GDC-0032)	preclinical	26589432	**B3**
MTAP	del.	breast	del.	sensitivity	5-Fluoropyrimidine	preclinical	26751376	**B3**
PIK3CA	C420R	colorectal	any (GoF)	response	aspirin in adjuvant setting	late trials	23094721	**B2**
PTEN	R130G	endometrial	any (LoF)	response	PARP inhibitors	case report	21468130, 20944090	**B2**
		breast	any (LoF)	sensitivity	ATM inhibitor	preclinical	27397505	**B3**
PIK3CA	C420R	breast	mut.	sensitivity	AZD-5363	clin. trials	28489509	**B2**
		colorectal	mut.	sensitivity	Cabozantinib	preclinical	25242168	**B3**
CDKN2A	del.	melanoma	loss	sensitivity	Flavopiridol	preclinical	12777976	**B3**
PTEN	R130G	breast	mut.	sensitivity	Ipatasertib	preclinical	23287563	**B3**
		endometrial	any (LoF)	no response	mTOR inhibitors	early trials	21788564, 23238879	**B2**
		melanoma	any (LoF)	resistance	PD1 inhibitors	early trials	26645196	**B2**
PIK3CA	C420R	endometrial	mut.	sensitivity	Ridaforolimus,Temsirolimus	clin. trials	24166148	**B2**
PTEN	R130G	unspecified	any (LoF)	response	sirolimus	early trials	ASCO 2013 (abstr 2532)	**B2**

Other genes: here you can find other genes that might be interesting to check (information from Target DB[3] and Meric-Bernstam list[4]). No level information is provided in this section.

[1] "No other genes found"

[3] Van Allen et al., Nat Med (2014), v3
[4] Meric-Bernstam et al., J Natl Cancer Inst (2015)

MTB Report - From somatic variants to treatment options

Department of Medical Statistics, University Medical Center Goettingen

September 27, 2017

PATIENT INFORMATION

**Patient ID**	MASTER-06	**Tissue Type**	?
**Gender**	Male	**Tumor Content (%)**	NA
**Disease**	Neuroendocrine tumor	**Number SNVs**	2703
**Previous Therapies**	Many chemotherapy cycles	**Number CNVs**	2114
**Tumor Board Decision**	mTOR missense mutations: mTOR inhibitor; PTPN12 missense mutation: Lapatinib, erlotinib; KIT missense mutation: imatinib, desatinib; LCK missense mutation: desatinib	**Number Fusions**	5

GENE-DRUG PREDICTIVE ASSOCIATIONS

Method: Somatic variants of the patient (mutations, amplifications, deletions, rearrangments) are searched in curated databases of predictive biomarkers (GKDB[1], CIViC[2]) and reported according to their clinical evidence (see Levels of Evidence). In the following two tables (SNVs and CNVs), basic information of the somatic variants with relevant clinical implications can be found:

Gene	Patient's Variant	Level of Evidence	Variant Freq.	Zygosity	Quality (Phred)
ABL1	A922T	B1 ,B2	0.09	het	0
APC	P411S	B3	0.41	het	225
ATM	V356I	B2 ,B3	1.00	hom	222
CDK12	E1292K	B3	0.44	het	225
CEBPA	G272D	B2	0.41	het	225
GNAQ	X68L	B2 ,B3	0.30	het	225
GNAS	G35E, A349T	B3	0.41	het	225
JAK1	E37K	B2 ,B3	0.48	het	225
JAK3	G442D	B3	0.15	het	6
KIT	A837T	B3	0.09	het	0
MTOR	P2490L, G332R	B2 ,B3	0.26	het	189
NF1	W777X	B2 ,B3	0.31	het	160
NOTCH1	L590F	B3	0.36	het	171
RAD50	D478N	B2	0.29	het	190
ROS1	R201K	B2 ,B3	0.78	het	225
SMAD4	G510E	B2	0.53	het	225
SMARCA4	S2F	B2	0.43	het	225
SMO	P306S	B2 ,B3	0.28	het	81
TSC2	D1380N	B2 ,B3	0.85	hom	225

Gene	Patient's Variant	Level of Evidence	Seg. Mean	Size (Mb)
CDKN2A	del.	B2 ,B3	-0.72	20.8
CDKN2B	del.	B3	-0.72	20.8
FBXW7	del.	B3	-0.69	42.3
GNAQ	X68L	B2 ,B3	0.58	15.4
MGMT	del.	B2	-0.66	45.0
MTAP	del.	B3	-0.72	20.8
SYK	ampl.	B3	0.55	4.7

Levels of Evidence: Findings are classified into 6 levels of evidence combining the **axis A-B** and the **axis 1-2-3**. Level A means evidence in the same cancer type. Level B means evidence in any other cancer type. On the 1-2-3 axis, level 1 means evidence supported by drug approval organizations or clinical guidelines, level 2 contains clinical evidence (clinical trials, case reports) and level 3 consists of preclinical evidence. The distribution of findings into levels is summarized in the right figure

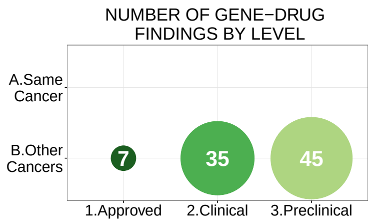

Table of Results: All the predicitve associations are detailed in this table. The results are sorted by 1) drug frequency, 2) levels of evidence (A1-B1-A2-B2-A3-B3). To allow a quick interpretation, the type of association (response, resistance) is colored (green, red) and new variants are gray and underlined.

Patient		Gene-Drug Associations						
Gene	Variant	Disease	Known Variant	Association	Drugs	Evidence	PMID	Level
NF1	W777X	neurosarcoma	any (LoF)	no response	everolimus + bevacizumab	early trials	ASCO 2016 (abstr 11053)	B2

[1]Dienstmann et al., Cancer Discov (2015), v19
[2]Griffith et al., Nat Genet (2017), version 01 June 2017

Patient		Gene-Drug Associations						
Gene	Variant	Disease	Known Variant	Association	Drugs	Evidence	PMID	Level
MTOR	P2490L G332R	angiosarcoma	I1973F, K1771R (GoF)	response	everolimus	case report	ASCO 2015 (abstr 11010),26859683	B2
		bladder	E2014K, E2419K, N1421D (GoF)	response	everolimus	case report	24625776,ASCO 2015 (abstr 11010)	B2
		gastric	K1771R, N1421D (GoF)	response	everolimus	case report	ASCO 2015 (abstr 11010),26859683	B2
		renal	I1973F, Q2223K (GoF)	response	everolimus	case report	26859683,24622468	B2
		bladder	mut.	sensitivity	Everolimus,Pazopanib	case report	24625776	B2
NF1	W777X	head and neck	D1644A (LoF)	response	everolimus	case report	26859683	B2
		hepatocellular	mut. (LoF)	response	everolimus + pazopanib	case report	24931142	B2
TSC2	D1380N	thyroid	any (LoF)	response	everolimus	case report	25295501	B2
		gastric	any (LoF)	response	everolimus	case report	26859683	B2
CDKN2A	del.	breast	loss	sensitivity	Letrozole,Palbociclib (PD0332991)	case report	26715889	B2
		bone ewing sarcoma	loss	sensitivity	Linsitinib,Palbociclib	preclinical	27286459	B3
		dermatofibrosarcoma	loss	sensitivity	Palbociclib	preclinical	25852058	B3
		ovarian	loss	sensitivity	Palbociclib (PD0332991)	preclinical	21278246	B3
		renal	loss	sensitivity	Palbociclib (PD0332991)	preclinical	23898052	B3
		melanoma	loss	sensitivity	Palbociclib (PD0332991)	preclinical	24495407	B3
CDKN2B	del.	renal	loss	sensitivity	Palbociclib (PD0332991)	preclinical	23898052	B3
		bone ewing sarcoma	loss	sensitivity	Palbociclib,Linsitinib	preclinical	27286459	B3
ABL1	A922T	CML	F359V/C/I, Y253H, E255K/V (GoF)	response	dasatinib, bosutinib, ponatinib	NCCN guidelines	21562040	B1
		ALL	F359V/C/I, Y253H, E255K/V (GoF)	response	dasatinib, ponatinib	NCCN guidelines	NCCN	B1
		CML	T315A, F317L/V/I/C (GoF)	response	nilotinib, bosutinib, ponatinib	NCCN guidelines	21562040	B1
		ALL	T315A, F317L/V/I/C, V299L (GoF)	response	nilotinib, ponatinib	NCCN guidelines	NCCN	B1
		CML	V299L (GoF)	response	nilotinib, ponatinib	NCCN guidelines	21562040	B1
		ALL	T315I (GoF)	response	ponatinib	NCCN guidelines	NCCN	B1
		CML	T315I (GoF)	response	ponatinib	NCCN guidelines	21562040	B1
CDKN2A	del.	melanoma	any (LoF)	response	CDK4/6 inhibitors	case report	ASCO 2013 (abstr 2500)	B2
		unspecified	any (LoF)	sensitivity	CDK4/6 inhibitors	preclinical	22471707	B3
		glioblastoma	any (LoF)	sensitivity	CDK4/6 inhibitors	preclinical	22586120, 22711607	B3
CDKN2B	del.	unspecified	any (LoF)	sensitivity	CDK4/6 inhibitors	preclinical	22471707	B3
		glioblastoma	any (LoF)	sensitivity	CDK4/6 inhibitors	preclinical	22711607	B3
TSC2	D1380N	angiomyolipoma	any (LoF)	response	mTOR inhibitors	early trials	23312829, 21525172, 20048174	B2
NF1	W777X	neurosarcoma	any (LoF)	sensitivity	mTOR inhibitors	preclinical	18483311, 20505189, 24509877	B3
		neurosarcoma	any (LoF)	sensitivity	mTOR inhibitors + HSP90 inhibitors	preclinical	21907929	B3
		glioblastoma	any (LoF)	sensitivity	mTOR inhibitors	preclinical	19573811	B3
MGMT	del.	glioblastoma	any (LoF)	increased benefit	temozolomide	late trials	15758010	B2
ATM	V356I	glioblastoma	any (LoF)	sensitivity	temozolomide	preclinical	23960094	B3
		glioblastoma	mut.	sensitivity	Temozolomide	preclinical	23960094	B3
		melanoma	mut.	sensitivity	Temozolomide	preclinical	23960094	B3

Patient Gene	Variant	Disease	Known Variant	Gene-Drug Association	Associations Drugs	Evidence	PMID	Level
SMO	P306S	basal cell carc.	mut.	resistance or non-response	Vismodegib	clin. trials	25759020	B2
		basal cell carc.	mut.	resistance or non-response	Vismodegib,LDE225	clin. trials	26546616	B2
		basal cell carc.	G497W, D473Y (GoF)	resistance	vismodegib	case report	25306392	B2
		medulloblastoma	D473H (GoF)	resistance	vismodegib	case report	19726788, 25759019	B2
NF1	W777X	melanoma	any (LoF)	resistance	selumetinib in BRAF mutant tumors	case report	23444215, 23288408	B2
		melanoma	any (LoF)	resistance	BRAF inhibitors in BRAF mutant tumor	preclinical	23288408, 24576830	B3
ATM	V356I	prostate	any (LoF)	response	PARP inhibitors	early trials	26510020	B2
		gastric	any (LoF)	response	PARP inhibitors	early trials	ENA 2014 (abstr 8LBA)	B2
CDK12	E1292K	ovarian	any (LoF)	sensitivity	PARP inhibitors	preclinical	24240700, 24554720	B3
FBXW7	del.	breast	(LoF)	sensitivity	Rapamycin (Sirolimus)	preclinical	18787170	B3
MTOR	P2490L G332R	unspecified	L1460P, S2215Y, R2505P (GoF)	sensitivity	rapamycin	preclinical	24631838	B3
NF1	W777X	melanoma	mut.	sensitivity	Rapamycin (Sirolimus),PD0325901	preclinical	23171796	B3
GNAS	G35E A349T	unspecified	R201 (GoF)	sensitivity	JAK inhibitors	preclinical	21835143	B3
JAK3	G442D	megakaryo. Leuk.	R657Q, I87T, Q501H (GoF)	sensitivity	JAK inhibitors	preclinical	18397343	B3
GNAQ	X68L	melanoma	mut.	sensitivity	JQ1	preclinical	26397223	B3
NF1	W777X	malignant peripheral nerve sheath tumor	loss	sensitivity	JQ1 Compound	preclinical	24373973	B3
		melanoma	any (LoF)	response	PD1 blockade	early trials	ASCO 2016 (abstr 105)	B2
JAK1	E37K	melanoma	any (LoF)	resistance	PD1 blockade	case report	27433843	B2
TSC2	D1380N D1380N	endometrial	any (GoF)	response	tensirolimus	case report	27016228	B2
GNAQ	X68L	melanoma	mut.	sensitivity	Trametinib	clin. trials	22805292	B2
NF1	W777X	melanoma	any (LoF)	sensitivity	trametinib	preclinical	24576830	B3
		melanoma	mut.	resistance or non-response	Vemurafenib	case report	23288408	B2
		melanoma	mut.	resistance or non-response	Vemurafenib	preclinical	23171796	B3
MTAP	del.	breast	del.	sensitivity	5-Fluoropyrimidine	preclinical	26751376	B3
CEBPA	G272D	AML	mut.	sensitivity	All-trans Retinoic Acid	clin. trials	19965647	B2
FBXW7	del.	unspecified	any (LoF)	resistance	anti-tubulin agents	preclinical	21368834	B3
SMO	P306S	basal cell carc.	mut.	sensitivity	Arsenic Trioxide,PSI	preclinical	25759020	B3
RAD50	D478N	unspecified	L1237F (LoF)	response	irinotecan plus CHK1/2 inhibitor in ATM deficient tumor	case report	24934408	B2
ATM	V356I	colorectal	any (LoF)	response	ATR inhibitors	case report	ENA 2015 (abstract A48)	B2
ABL1	A922T	ALL	T315I (GoF)	response	AURK inhibitors + BCR-ABL1 inhibitors	case report	22772060	B2
		ALL	T315I (GoF)	response	axitinib	case report	25686603	B2
NF1	W777X	melanoma	mut.	sensitivity	VTX-11e,AZ628	preclinical	23288408	B3
ROS1	R201K	lung (adeno)	G2032R (GoF)	sensitivity	cabozantinib	preclinical	25351743	B3
SMAD4	G510E	colorectal	mut.	resistance or non-response	Panitumumab,Cetuximab	clin. trials	26508446	B2
ATM	V356I	bladder	any (LoF)	response	cisplatin	early trials	26238431	B2
ROS1	R201K	lung (adeno)	G2032R (GoF)	resistance	crizotinib	case report	23724914, 25688157	B2
ATM	V356I	anaplast. Large-cell lymph.	any (LoF)	sensitivity	DNA-PKc inhibitors	preclinical	23761041	B3
SMARCA4	S2F	ovarian rhabdoid	any (LoF)	response	EZH2 inhibitor	case report	ESMO 2015 (abstract 302)	B2
CDKN2A	del.	melanoma	loss	sensitivity	Flavopiridol	preclinical	12777976	B3
APC	P411S	colorectal	mut.	sensitivity	G007-LK	preclinical	23539443	B3
NOTCH1	L590F	mantle cell lymphoma	any (GoF)	sensitivity	Gamma secretase inhibitors	preclinical	22210878	B3

Patient		Gene-Drug Associations						
Gene	Variant	Disease	Known Variant	Association	Drugs	Evidence	PMID	Level
KIT	A837T	gastric (stromal)	mut.	sensitivity	Pictilisib,Imatinib	preclinical	23231951	B3
APC	P411S	colorectal	mut.	sensitivity	JW55	preclinical	22440753	B3
NF1	W777X	neurosarcoma	any (LoF)	sensitivity	MEK inhibitors	preclinical	23221341	B3
ATM	V356I		mut.	sensitivity	Olaparib	preclinical	20739657	B3
JAK1	E37K	ALL	S646F (GoF)	sensitivity	ruxolitinib	preclinical	22955920	B3
TSC2	D1380N	lymphangio-leiomyomatosis	any (LoF)	sensitivity	SRC inhibitors	preclinical	24691995	B3
SYK	ampl.	CLL	ampl. (GoF)	sensitivity	SYK inhibitors	preclinical	16409295, 19549911	B3
APC	P411S	colorectal	any (LoF)	sensitivity	tankyrase inhibitors	preclinical	22440753, 23539443	B3

Other genes: here you can find other genes that might be interesting to check (information from Target DB[3] and Meric-Bernstam list[4]). No level information is provided in this section.

Patient		Drug-Gene Interactions		
Gene	Variant	Known Variant	Description	Drugs
NOTCH3	L1185F	Mutation; Amplification; Rearrangement		Treatment with GSIs
CREBBP	A237T	Biallelic Inactivation	Biallelic inactivation is Prognostic / Diagnostic in leukemia	

[3]Van Allen et al., Nat Med (2014), v3
[4]Meric-Bernstam et al., J Natl Cancer Inst (2015)

MTB Report - From somatic variants to treatment options

Department of Medical Statistics, University Medical Center Goettingen

September 27, 2017

PATIENT INFORMATION

**Patient ID**	MASTER-07	**Tissue Type**	?
**Gender**	Male	**Tumor Content (%)**	70
**Disease**	Gastric cardia metastasis IHC: neuroendocine	**Number SNVs**	657
**Previous Therapies**	Chemotherapy	**Number CNVs**	425
**Tumor Board Decision**	ERBB3 amplification, RAF1 missense mutation, MTOR missense mutation : mTOR inhibitor	**Number Fusions**	5

GENE-DRUG PREDICTIVE ASSOCIATIONS

Method: Somatic variants of the patient (mutations, amplifications, deletions, rearrangments) are searched in curated databases of predictive biomarkers (GKDB[1], CIViC[2]) and reported according to their clinical evidence (see Levels of Evidence). In the following two tables (SNVs and CNVs), basic information of the somatic variants with relevant clinical implications can be found:

Gene	Patient's Variant	Level of Evidence	Variant Freq.	Zygosity	Quality (Phred)
AR	A417V	B2 ,B3	0.53	het	131
ARID1A	G1450fs	B2 ,B3	0.00	NA	2962
ERBB3	V104M	B2 ,B3	0.37	het	225
MTOR	E1485G	A2 ,B2 ,B3	0.20	het	27
PBRM1	1092-1092del	B3	0.00	NA	2958
RAF1	S259P	B3	0.22	het	136
RUNX1	A329T	B2	0.31	het	167
TP53	R175H	A2 ,B2 ,B3	0.39	het	225

Gene	Patient's Variant	Level of Evidence	Seg. Mean	Size (Mb)
CDK4	ampl.	B2	0.99	3.1
ERBB3	V104M,ampl.	B2 ,B3	0.99	3.1

Levels of Evidence: Findings are classified into 6 levels of evidence combining the **axis A-B** and the **axis 1-2-3**. Level A means evidence in the same cancer type. Level B means evidence in any other cancer type. On the 1-2-3 axis, level 1 means evidence supported by drug approval organizations or clinical guidelines, level 2 contains clinical evidence (clinical trials, case reports) and level 3 consists of preclinical evidence. The distribution of findings into levels is summarized in the right figure

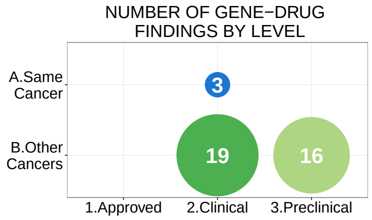

Table of Results: All the predicitve associations are detailed in this table. The results are sorted by 1) drug frequency, 2) levels of evidence (A1-B1-A2-B2-A3-B3). To allow a quick interpretation, the type of association (response, resistance) is colored (green, red) and new variants are gray and underlined.

Patient Gene	Patient Variant	Disease	Known Variant	Gene-Drug Association	Associations Drugs	Evidence	PMID	Level
MTOR	E1485G	gastric	K1771R, N1421D (GoF)	response	everolimus	case report	ASCO 2015 (abstr 11010),26859683	A2
		angiosarcoma	I1973F, K1771R (GoF)	response	everolimus	case report	ASCO 2015 (abstr 11010),26859683	B2
		bladder	E2014K, E2419K, N1421D (GoF)	response	everolimus	case report	24625776,ASCO 2015 (abstr 11010)	B2
		renal	I1973F, Q2223K (GoF)	response	everolimus	case report	26859683,24622468	B2
		bladder	mut.	sensitivity	Everolimus,Pazopanib	case report	24625776	B2
ERBB3	V104M	bladder	V104M, R103G, G284R	sensitivity	Afatinib	clin. trials	27044931	B2
		bladder	G284R, V104M, R103G (GoF)	response	afatinib	case report	27044931	B2
		bladder	G284R, R103G (GoF)	response	afatinib	case report	ASCO 2015 (abstr e15516)	B2

[1] Dienstmann et al., Cancer Discov (2015), v19
[2] Griffith et al., Nat Genet (2017), version 01 June 2017

Patient		Gene-Drug Associations						
Gene	Variant	Disease	Known Variant	Association	Drugs	Evidence	PMID	Level
ARID1A	G1450fs	ovarian	any (LoF)	sensitivity	EZH2 inhibitor	preclinical	25686104	B3
		unspecified	mut. (LoF)	sensitivity	EZH2 inhibitors in RAS wt	preclinical	26552009	B3
PBRM1	1092-1092del	unspecified	any (LoF)	sensitivity	EZH2 inhibitor in RAS wt	preclinical	26552009	B3
ARID1A	G1450fs	breast	mut. (LoF)	resistance	trastuzumab (ANXA1 high)	early trials	27172896	B2
ERBB3	V104M	unspecified	Q809R (GoF)	sensitivity	trastuzumab, lapatinib, PI3K pathway inhibitors + MEK inhibitors	preclinical	23680147	B3
		unspecified	P262H, G284R (GoF)	sensitivity	trastuzumab, pertuzumab, lapatinib, anti-HER3 mAbs, PI3K pathway inhibitors + MEK inhibitors	preclinical	23680147	B3
RAF1	S259P	melanoma	S257P, P261P, G361A (GoF)	resistance	BRAF inhibitors in BRAF mutant tumors	preclinical	23737487	B3
TP53	R175H	AML	mut. (LoF)	response	decitabine	early trials	27959731	B2
		MDS	mut. (LoF)	response	decitabine	early trials	27959731	B2
		lung	mut.	resistance or non-response	Docetaxel	preclinical	22425996	B3
		lung	mut.	sensitivity	Docetaxel,Selumetinib (AZD6244)	preclinical	22425996	B3
		bladder	mut. (LoF)	sensitivity	mitomycin C, gemcitabine, doxorubicin	preclinical	27397505	B3
		breast	R175H, mut.	sensitivity	Doxorubicin	preclinical	22698404	B3
		ovarian	any (GoF)	response	WEE1 inhibitors + carboplatin	early trials	ASCO2015 (abstr 2507)	B2
		head and neck	any (LoF)	sensitivity	WEE1 inhibitors	preclinical	25125259	B3
		breast	mut. (LoF)	resistance	CDK4/CDK6 inhibitor abemaciclib	early trials	27217383	B2
AR	A417V	prostate	mut.	resistance or non-response	Flutamide,Nilutamide,Bicalutamide,Cyproterone Acetate	clin. trials	26000489	B2
TP53	R175H	CLL	mut.	sensitivity	Alemtuzumab	clin. trials	14726385	B2
ERBB3	ampl.	colorectal	ampl. (GoF)	resistance	anti-EGFR mAbs	early trials	25520391	B2
CDK4	ampl.	liposarcoma	ampl. (GoF)	response	CDK4 inhibitors (tumors with RB expression)	early trials	23569312	B2
TP53	R175H	gastric	mut.	sensitivity	Chemotherapy	clin. trials	24740294	A2
			mut. (LoF)	resistance	cisplatin	early trials	27646943	B2
RUNX1	A329T	AML	mut.	resistance or non-response	Cytarabine	clin. trials	21343560	B2
TP53	R175H	gastric	R273C, Y220C, R175H, R282L, R213P, mut.	sensitivity	EAP Protocol	case report	14514923	A2
AR	A417V	prostate	F877L/T878A coexisting (GoF)	sensitivity	enzalutamide	preclinical	27196756	B3
TP53	R175H	unspecified	R248Q, R175H (GoF)	sensitivity	HSP90 inhibitors	preclinical	26009011	B3
ARID1A	G1450fs	unspecified	mut. (LoF)	sensitivity	PARP inhibitors	preclinical	26069190	B3
CDK4	ampl.	liposarcoma	ampl.	sensitivity	PD0332991	clin. trials	23569312	B2
TP53	R175H	thymic lymphoma	any (LoF)	sensitivity	pramlintide	preclinical	25409149	B3
MTOR	E1485G	unspecified	L1460P, S2215Y, R2505P (GoF)	sensitivity	rapamycin	preclinical	24631838	B3

Other genes: here you can find other genes that might be interesting to check (information from Target DB[3] and Meric-Bernstam list[4]). No level information is provided in this section.

Patient		Drug-Gene Interactions		
Gene	Variant	Known Variant	Description	Drugs
MAP3K4	T1055M	Mutation; Amplification		Treatment with JNK1 inhibitor
MAPK8	E1103K	Mutation; Amplification		Treatment with JNK1 inhibitor

[3]Van Allen et al., Nat Med (2014), v3
[4]Meric-Bernstam et al., J Natl Cancer Inst (2015)

MTB Report - From somatic variants to treatment options

Department of Medical Statistics, University Medical Center Goettingen

September 27, 2017

PATIENT INFORMATION

**Patient ID**	MASTER-08	**Tissue Type**	?
**Gender**	Male	**Tumor Content (%)**	60
**Disease**	metastasis cholagiocarcinoma	**Number SNVs**	28
**Previous Therapies**	Erlotinib	**Number CNVs**	1011
**Tumor Board Decision**	ERRFI1 stopgain is promising (EGFR/ERBB2-4 negative regulation) : Erlotinib	**Number Fusions**	1

GENE-DRUG PREDICTIVE ASSOCIATIONS

Method: Somatic variants of the patient (mutations, amplifications, deletions, rearrangments) are searched in curated databases of predictive biomarkers (GKDB[1], CIViC[2]) and reported according to their clinical evidence (see Levels of Evidence). In the following two tables (SNVs and CNVs), basic information of the somatic variants with relevant clinical implications can be found:

Gene	Patient's Variant	Level of Evidence	Variant Freq.	Zygosity	Quality (Phred)
ARID1A	R1276X	**B2** ,B3	0.31	het	225

Gene	Patient's Variant	Level of Evidence	Seg. Mean	Size (Mb)
FGFR1	ampl.	**B2** ,B3	0.68	38.4
MCL1	ampl.	B3	0.79	19.8

Levels of Evidence: Findings are classified into 6 levels of evidence combining the **axis A-B** and the **axis 1-2-3**. Level A means evidence in the same cancer type. Level B means evidence in any other cancer type. On the 1-2-3 axis, level 1 means evidence supported by drug approval organizations or clinical guidelines, level 2 contains clinical evidence (clinical trials, case reports) and level 3 consists of preclinical evidence. The distribution of findings into levels is summarized in the right figure

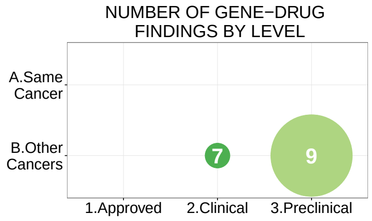

Table of Results: All the predicitve associations are detailed in this table. The results are sorted by 1) drug frequency, 2) levels of evidence (A1-B1-A2-B2-A3-B3). To allow a quick interpretation, the type of association (response, resistance) is colored (green, red) and new variants are gray and underlined.

Patient		Gene-Drug		Associations				
**Gene**	**Variant**	**Disease**	**Known Variant**	**Association**	**Drugs**	**Evidence**	**PMID**	**Level**
FGFR1	ampl.	breast	ampl.	sensitivity	BGJ-398	clin. trials	27870574	**B2**
		bladder	ampl.	sensitivity	BGJ-398	case report	27870574	**B2**
ARID1A	R1276X	ovarian	any (LoF)	sensitivity	EZH2 inhibitor	preclinical	25686104	B3
		unspecified	mut. (LoF)	sensitivity	EZH2 inhibitors in RAS wt	preclinical	26552009	B3
FGFR1	ampl.	lung (squa.)	ampl. (GoF)	response	FGFR inhibitors	early trials	AACR 2012 (abstract LB-122), AACR 2013 (abstract LB-145)	**B2**
		breast	ampl. (GoF)	response	FGFR inhibitors	early trials	25193991	**B2**
		lung	ampl.	sensitivity	PD173074	preclinical	21160078	B3
		lung	ampl.	sensitivity	PD173074	preclinical	21666749	B3
		bone ewing sarcoma	ampl.	sensitivity	Ponatinib	preclinical	26179511	B3
		breast	ampl.	sensitivity	Ponatinib	preclinical	22238366	B3
		breast	ampl.	resistance or non-response	4-hydroxytamoxifen	preclinical	20179196	B3
MCL1	ampl.	unspecified	ampl. (GoF)	resistance	anti-tubulin agents	preclinical	21368834	B3
ARID1A	R1276X	breast	mut. (LoF)	resistance	trastuzumab (ANXA1 high)	early trials	27172896	**B2**
FGFR1	ampl.	lung	ampl.	sensitivity	BGJ398	clin. trials	27870574	**B2**
		breast	ampl.	sensitivity	Dovitinib	clin. trials	23658459	**B2**
ARID1A	R1276X	unspecified	mut. (LoF)	sensitivity	PARP inhibitors	preclinical	26069190	B3

Other genes: here you can find other genes that might be interesting to check (information

[1]Dienstmann et al., Cancer Discov (2015), v19
[2]Griffith et al., Nat Genet (2017), version 01 June 2017

from Target DB[3] and Meric-Bernstam list[4]). No level information is provided in this section.

Patient		Drug-Gene Interactions		
Gene	Variant	Known Variant	Description	Drugs
ERRFI1	R199X	Biallelic Inactivation	Deletion may predict sensitivity to EGFR inhibitors	Erlotinib, Gefitinib, EGFR Inhibitors

[3]Van Allen et al., Nat Med (2014), v3
[4]Meric-Bernstam et al., J Natl Cancer Inst (2015)

MTB Report - From somatic variants to treatment options

Department of Medical Statistics, University Medical Center Goettingen

September 27, 2017

PATIENT INFORMATION

**Patient ID**	MASTER-09	**Tissue Type**	?
**Gender**	Female	**Tumor Content (%)**	NA
**Disease**	Clear cell sarcoma, bone metastasis	**Number SNVs**	11
**Previous Therapies**	-	**Number CNVs**	1
**Tumor Board Decision**	NTRK3 nissense mutation: lestaurtinib, midostaurin	**Number Fusions**	20

GENE-DRUG PREDICTIVE ASSOCIATIONS

Method: Somatic variants of the patient (mutations, amplifications, deletions, rearrangments) are searched in curated databases of predictive biomarkers (GKDB[1], CIViC[2]) and reported according to their clinical evidence (see Levels of Evidence). In the following two tables (SNVs and CNVs), basic information of the somatic variants with relevant clinical implications can be found:

Levels of Evidence: Findings are classified into 6 levels of evidence combining the **axis A-B** and the **axis 1-2-3**. Level A means evidence in the same cancer type. Level B means evidence in any other cancer type. On the 1-2-3 axis, level 1 means evidence supported by drug approval organizations or clinical guidelines, level 2 contains clinical evidence (clinical trials, case reports) and level 3 consists of preclinical evidence. The distribution of findings into levels is summarized in the right figure

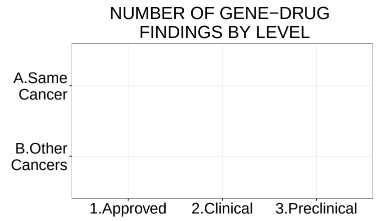

Table of Results: All the predicitve associations are detailed in this table. The results are sorted by 1) drug frequency, 2) levels of evidence (A1-B1-A2-B2-A3-B3). To allow a quick interpretation, the type of association (response, resistance) is colored (green, red) and new variants are gray and underlined.

Other genes: here you can find other genes that might be interesting to check (information from Target DB[3] and Meric-Bernstam list[4]). No level information is provided in this section.

**Patient**		**Drug-Gene Interactions**		
**Gene**	**Variant**	**Known Variant**	**Description**	**Drugs**
RARA	F189S	Rearrangement; mutation	Transloactions predict sensitivity to ATRA and arsenic	ATRA, Arsenic
EWSR1	EWSR1 fusion	Rearrangement	Diagnostic in Ewing sarcoma	
RUNX1	RUNX1 fusion	Rearrangement	May be prognostic / diagnostic in some hematologic malginancies.	

[1]Dienstmann et al., Cancer Discov (2015), v19
[2]Griffith et al., Nat Genet (2017), version 01 June 2017
[3]Van Allen et al., Nat Med (2014), v3
[4]Meric-Bernstam et al., J Natl Cancer Inst (2015)

MTB Report - From somatic variants to treatment options

Department of Medical Statistics, University Medical Center Goettingen

September 28, 2017

PATIENT INFORMATION

**Patient ID**	MASTER-10	**Tissue Type**	?
**Gender**	Male	**Tumor Content (%)**	70
**Disease**	Urothelial carcinoma. Neu spine tumors, and lungs	**Number SNVs**	7
**Previous Therapies**	-	**Number CNVs**	5
**Tumor Board Decision**	BRAF + HRAS missense mutations : MEK-Inhibitor against risk of paradoxical activation of ERK after BRAF-inhibitors or Sorafenib	**Number Fusions**	1

GENE-DRUG PREDICTIVE ASSOCIATIONS

Method: Somatic variants of the patient (mutations, amplifications, deletions, rearrangments) are searched in curated databases of predictive biomarkers (GKDB[1], CIViC[2]) and reported according to their clinical evidence (see Levels of Evidence). In the following two tables (SNVs and CNVs), basic information of the somatic variants with relevant clinical implications can be found:

Gene	Patient's Variant	Level of Evidence	Variant Freq.	Zygosity	Quality (Phred)
BRAF	F595L	B2 ,B3	0.11	het	57
HRAS	Q61R	B2 ,B3	0.08	het	0
RAC1	D63N	B2	0.04	het	0

Gene	Patient's Variant	Level of Evidence	Seg. Mean	Size (Mb)

Levels of Evidence: Findings are classified into 6 levels of evidence combining the **axis A-B** and the **axis 1-2-3**. Level A means evidence in the same cancer type. Level B means evidence in any other cancer type. On the 1-2-3 axis, level 1 means evidence supported by drug approval organizations or clinical guidelines, level 2 contains clinical evidence (clinical trials, case reports) and level 3 consists of preclinical evidence. The distribution of findings into levels is summarized in the right figure

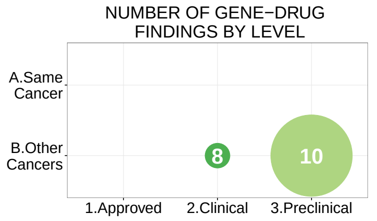

Table of Results: All the predicitve associations are detailed in this table. The results are sorted by 1) drug frequency, 2) levels of evidence (A1-B1-A2-B2-A3-B3). To allow a quick interpretation, the type of association (response, resistance) is colored (green, red) and new variants are gray and underlined.

Patient				Gene-Drug	Associations			
**Gene**	**Variant**	**Disease**	**Known Variant**	**Association**	**Drugs**	**Evidence**	**PMID**	**Level**
BRAF	F595L	melanoma	K601R (GoF)	response	MEK inhibitors	case report	23248257	B2
		melanoma	L597R (GoF)	response	trametinib, MEK inhibitors	case report	22805292, 22798288	B2
		lung (adeno)	G469A (GoF)	sensitivity	EGFR TKIs + MEK inhibitors in EGFR mutant	preclinical	22773810	B3
HRAS	Q61R	AML	any (GoF)	sensitivity	MEK inhibitors +/- mTOR inhibitors	preclinical	22399013, 22507781	B3
		cervical	any (GoF)	sensitivity	PI3K pathway inhibitors + MEK inhibitors	preclinical	15950068	B3
		breast	mut.	sensitivity	Selumetinib (AZD6244),Binimetinib (MEK162),PD0325901,Everolimus,AZD8055	preclinical	26544513	B3
BRAF	F595L	melanoma	L597R (GoF)	response	BRAF inhibitors	case report	23715574	B2
RAC1	D63N	melanoma	P29S (GoF)	resistance	BRAF inhibitors in BRAF mutant tumors	case report	25056119	B2
BRAF	F595L	lung (adeno)	G469A (GoF)	resistance	EGFR TKIs	case report	22773810	B2
		colorectal	mut.	sensitivity	Cetuximab,Panitumumab	clin. trials	25673558	B2
		colorectal	mut.	resistance or non-response	Cetuximab	preclinical	22586653	B3
		lung (adeno)	Y472C reduced kinase activity	response	dasatinib	case report	22649091	B2
		lung (adeno)	G466V reduced kinase activity	sensitivity	dasatinib	preclinical	22649091	B3
HRAS	Q61R	cervical	any (GoF)	sensitivity	mTOR inhibitors	preclinical	22345164	B3
BRAF	F595L	melanoma	D594G (GoF) (low activity)	sensitivity	sorafenib	preclinical	18794803	B3

[1] Dienstmann et al., Cancer Discov (2015), v19

[2] Griffith et al., Nat Genet (2017), version 01 June 2017

Patient				Gene-Drug	Associations			
**Gene**	**Variant**	**Disease**	**Known Variant**	**Association**	**Drugs**	**Evidence**	**PMID**	**Level**
		melanoma	G469E (GoF) (low activity)	sensitivity	sorafenib	preclinical	18794803	**B3**
		breast	mut.	sensitivity	Trametinib	preclinical	22169769	**B3**
HRAS	Q61R	unspecified	any (GoF)	sensitivity	tipifarnib	early trials	NCT02383927	**B2**

Other genes: here you can find other genes that might be interesting to check (information from Target DB[3] and Meric-Bernstam list[4]). No level information is provided in this section.

[1] "No other genes found"

[3]Van Allen et al., Nat Med (2014), v3
[4]Meric-Bernstam et al., J Natl Cancer Inst (2015)

MTB Report - From somatic variants to treatment options

Department of Medical Statistics, University Medical Center Goettingen

September 27, 2017

PATIENT INFORMATION

**Patient ID**	MASTER-11	**Tissue Type**	?
**Gender**	Female	**Tumor Content (%)**	80
**Disease**	Adeno-CUP (from Pulmonaly Adenocarci-noma)	**Number SNVs**	70
**Previous Therapies**	-	**Number CNVs**	133
**Tumor Board Decision**	EGFR p.745-750del: Erlotinib	**Number Fusions**	1

GENE-DRUG PREDICTIVE ASSOCIATIONS

Method: Somatic variants of the patient (mutations, amplifications, deletions, rearrangments) are searched in curated databases of predictive biomarkers (GKDB[1], CIViC[2]) and reported according to their clinical evidence (see Levels of Evidence). In the following two tables (SNVs and CNVs), basic information of the somatic variants with relevant clinical implications can be found:

Gene	Patient's Variant	Level of Evidence	Variant Freq.	Zygosity	Quality (Phred)
ABL1	D233N	**B1** ,**B2**	0.09	het	0
ATM	A749V	**B2** ,**B3**	0.07	het	0
EGFR	745-750del	**B1** ,**B2** ,**B3**	0.40	het	2927

Gene	Patient's Variant	Level of Evidence	Seg. Mean	Size (Mb)
CDK6	ampl.	**B3**	2.07	1.6
EGFR	745-750del,ampl.	**B1** ,**B2** ,**B3**	0.93	2.2
FOXA1	ampl.	**B3**	2.81	3.6
MYC	ampl.	**B3**	0.85	0.8

Levels of Evidence: Findings are classified into 6 levels of evidence combining the **axis A-B** and the **axis 1-2-3**. Level A means evidence in the same cancer type. Level B means evidence in any other cancer type. On the 1-2-3 axis, level 1 means evidence supported by drug approval organizations or clinical guidelines, level 2 contains clinical evidence (clinical trials, case reports) and level 3 consists of preclinical evidence. The distribution of findings into levels is summarized in the right figure

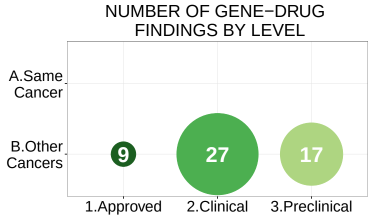

Table of Results: All the predicitve associations are detailed in this table. The results are sorted by 1) drug frequency, 2) levels of evidence (A1-B1-A2-B2-A3-B3). To allow a quick interpretation, the type of association (response, resistance) is colored (green, red) and new variants are gray and underlined.

Patient		Gene-Drug Associations						
**Gene**	**Variant**	**Disease**	**Known Variant**	**Association**	**Drugs**	**Evidence**	**PMID**	**Level**
ABL1	D233N	CML	F359V/C/I, Y253H, E255K/V (GoF)	response	dasatinib, bosutinib, ponatinib	NCCN guidelines	21562040	**B1**
		ALL	F359V/C/I, Y253H, E255K/V (GoF)	response	dasatinib, ponatinib	NCCN guidelines	NCCN	**B1**
		CML	T315A, F317L/V/I/C (GoF)	response	nilotinib, bosutinib, ponatinib	NCCN guidelines	21562040	**B1**
		ALL	T315A, F317L/V/I/C, V299L (GoF)	response	nilotinib, ponatinib	NCCN guidelines	NCCN	**B1**
		CML	V299L (GoF)	response	nilotinib, ponatinib	NCCN guidelines	21562040	**B1**
		ALL	T315I (GoF)	response	ponatinib	NCCN guidelines	NCCN	**B1**
		CML	T315I (GoF)	response	ponatinib	NCCN guidelines	21562040	**B1**
EGFR	745-750del	lung	exon 19 p.729-761 (GoF)	response	erlotinib, afatinib	NCCN guidelines	22190593	**B1**
		lung	exon 19 p.729-761 (GoF)	response	erlotinib, afatinib, gefitinib	FDA-approved	FDA	**B1**

[1]Dienstmann et al., Cancer Discov (2015), v19
[2]Griffith et al., Nat Genet (2017), version 01 June 2017

Patient		Gene-Drug Associations						
Gene	Variant	Disease	Known Variant	Association	Drugs	Evidence	PMID	Level
		lung	exon 19 p.729-761 (GoF)	response	afatinib	late trials	22753918, 25589191	B2
		lung	mut.	sensitivity	Afatinib	clin. trials	27083334	B2
		lung	exon 19 p.729-761 (GoF)	response	afatinib + cetuximab	early trials	ESMO 2012 (abstr 1289)	B2
		lung	exon 19 p.729-761 (GoF)	response	afatinib + cetuximab	early trials	ESMO 2012 (abstr 1289)	B2
		colorectal	ampl., expression	sensitivity	Cetuximab	clin. trials	18794099,18003960,14993230	B2
		colorectal	ampl.	sensitivity	Panitumumab,Cetuximab	clin. trials	24653627	B2
		head and neck	ampl.	sensitivity	PLATINUM,Cetuximab,5-fluorouracil	clin. trials	21048039	B2
		lung	L858R, ampl., G719S	sensitivity	Gefitinib,Erlotinib	clin. trials	24457318,2672208[illegible],26124334	B2
		lung	ampl. (GoF)	no response	erlotinib	early trials	ASCO 2015 (abstr e19028)	B2
		lung	V769-770insASV (GoF)	response	erlotinib	case report	26773740, 23328547	B2
		esophageal	ampl. (GoF)	response	gefitinib	late trials	24950987	B2
		head and neck	ampl. (GoF)	no response	gefitinib	early trials	21274259, 22261807	B2
		head and neck	delL747-P753insS (GoF)	no response	gefitinib	case report	21274259	B2
		lung	exon 19 p.729-761 (GoF)	response	irreversible EGFR TKIs	late trials	22753918	B2
		lung	exon 20 p.762-823 (GoF)	decreased sensitivity	first-generation and irreversible EGFR TKIs	late trials	21764376, 26773740, 26051236	B2
		colorectal	ampl. (GoF)	response	anti-EGFR mAbs	late trials	18794099, 17664472	B2
		lung	overexpression, ampl.	sensitivity	EGFR Inhibitor	clin. trials	22056021,2643980[illegible],20826716	B2
		head and neck	ampl. (GoF)	response	EGFR inhibitors	case report	26763254	B2
ATM	A749V	glioblastoma	any (LoF)	sensitivity	temozolomide	preclinical	23960094	B3
		glioblastoma	mut.	sensitivity	Temozolomide	preclinical	23960094	B3
		melanoma	mut.	sensitivity	Temozolomide	preclinical	23960094	B3
MYC	ampl.	colorectal	ampl. (GoF)	sensitivity	temozolomide	preclinical	27397505	B3
EGFR	745-750del	lung	exon 19 p.729-761 (GoF)	response	HSP90 inhibitors	early trials	ESMO 2012 (abstr 4380)	B2
		lung	exon 19 p.729-761 (GoF)	response	HSP90 inhibitors	early trials	ESMO 2012 (abstr 4380)	B2
		lung	exon 20 p.762-823 (GoF)	response	HSP90 inhibitors	early trials	ASCO 2014 (abstr 8015)	B2
		lung	exon 19 p.729-761 (GoF)	sensitivity	MEK inhibitors (alone or in combination)	preclinical	23102728	B3
		lung	exon 19 p.729-761 (GoF)	sensitivity	MEK inhibitors (alone or in combination)	preclinical	23102728	B3
MYC	ampl.	myeloma	ampl. (GoF)	sensitivity	BET inhibitors	preclinical	21889194	B3
		neuroblastoma	ampl. (GoF)	sensitivity	BET inhibitors	preclinical	23430699	B3
EGFR	745-750del	lung	exon 20 p.762-823 (GoF)	sensitivity	osimertinib	preclinical	26515464	B3
		lung	ampl.	resistance or non-response	Osimertinib,Rociletinib	preclinical	28202511	B3
ATM	A749V	prostate	any (LoF)	response	PARP inhibitors	early trials	26510020	B2
		gastric	any (LoF)	response	PARP inhibitors	early trials	ENA 2014 (abstr 8LBA)	B2
		colorectal	any (LoF)	response	ATR inhibitors	case report	ENA 2015 (abstract A48)	B2
ABL1	D233N	ALL	T315I (GoF)	response	AURK inhibitors + BCR-ABL1 inhibitors	case report	22772060	B2
		ALL	T315I (GoF)	response	axitinib	case report	25686603	B2
FOXA1	ampl.	colorectal	ampl. (GoF)	sensitivity	BCL2 inhibitors	preclinical	27397505	B3
CDK6	ampl.	unspecified	ampl. (GoF)	sensitivity	CDK6 inhibitors	preclinical	22471707	B3
MYC	ampl.	neuroblastoma	ampl. (GoF)	sensitivity	CDK7 inhibitors	preclinical	25416950	B3
ATM	A749V	bladder	any (LoF)	response	cisplatin	early trials	26238431	B2
		anaplast. Large-cell lymph.	any (LoF)	sensitivity	DNA-PKc inhibitors	preclinical	23761041	B3

Patient		Gene-Drug Associations							
**Gene**	**Variant**	**Disease**	**Known Variant**	**Association**	**Drugs**	**Evidence**	**PMID**	**Level**
MYC	ampl.	neuroblastoma	ampl. (GoF)	sensitivity	FACT inhibitor	preclinical	26537256	B3
ATM	A749V		mut.	sensitivity	Olaparib	preclinical	20739657	B3
MYC	ampl.	prostate	ampl. (GoF)	sensitivity	PIM inhibitors	preclinical	25505253	B3

Other genes: here you can find other genes that might be interesting to check (information from Target DB[3] and Meric-Bernstam list[4]). No level information is provided in this section.

**Patient**		**Drug-Gene Interactions**		
**Gene**	**Variant**	**Known Variant**	**Description**	**Drugs**
NKX2-1	amp	Amplification	Diagnostic in lung cancer	

[3]Van Allen et al., Nat Med (2014), v3
[4]Meric-Bernstam et al., J Natl Cancer Inst (2015)
